# IDH1 mutations induce organelle defects via dysregulated phospholipids

**DOI:** 10.1038/s41467-020-20752-6

**Published:** 2021-01-27

**Authors:** Adrian Lita, Artem Pliss, Andrey Kuzmin, Tomohiro Yamasaki, Lumin Zhang, Tyrone Dowdy, Christina Burks, Natalia de Val, Orieta Celiku, Victor Ruiz-Rodado, Elena-Raluca Nicoli, Michael Kruhlak, Thorkell Andresson, Sudipto Das, Chunzhang Yang, Rebecca Schmitt, Christel Herold-Mende, Mark R. Gilbert, Paras N. Prasad, Mioara Larion

**Affiliations:** 1 Neuro-Oncology Branch, National Cancer Institute, Center for Cancer Research, National Institutes of Health, Bethesda, MD USA; 2 Institute for Lasers, Photonics and Biophotonics, University at Buffalo, State University of New York, Buffalo, NY 14260 USA; 3 Advanced Cytometry Instrumentation Systems, LLC, Buffalo, NY 14260 USA; 4 Electron Microscopy Laboratory, Frederick National Laboratory for Cancer Research, Center for Cancer Research, National Cancer Institute, Frederick, MD 21702 USA; 5 Center for Molecular Microscopy, Center for Cancer Research, National Cancer Institute, Frederick, MD 21702 USA; 6 Cancer Research Technology Program, Frederick National Laboratory for Cancer Research, Frederick, MD 21701 USA; 7 Undiagnosed Diseases Program, National Human Genome Research Institute (NHGRI), National Institutes of Health (NIH), Bethesda, MD 20892 USA; 8 Laboratory of Cancer Biology and Genetics, National Cancer Institute, National Institutes of Health (NIH), Bethesda, MD 20892 USA; 9 Protein Characterization Laboratory of the Cancer Research Technology Program (CRTP), National Cancer Institute, Frederick, MD 21702 USA; 10 Division of Neurosurgical Research, Department of Neurosurgery, University Hospital Heidelberg, Heidelberg, Germany

**Keywords:** Cancer metabolism, Cancer metabolism

## Abstract

Infiltrating gliomas are devastating and incurable tumors. Amongst all gliomas, those harboring a mutation in isocitrate dehydrogenase 1 mutation (IDH1^*mut*^) acquire a different tumor biology and clinical manifestation from those that are IDH1^WT^. Understanding the unique metabolic profile reprogrammed by IDH1 mutation has the potential to identify new molecular targets for glioma therapy. Herein, we uncover increased monounsaturated fatty acids (MUFA) and their phospholipids in endoplasmic reticulum (ER), generated by IDH1 mutation, that are responsible for Golgi and ER dilation. We demonstrate a direct link between the IDH1 mutation and this organelle morphology via D-2HG-induced stearyl-CoA desaturase (SCD) overexpression, the rate-limiting enzyme in MUFA biosynthesis. Inhibition of IDH1 mutation or SCD silencing restores ER and Golgi morphology, while D-2HG and oleic acid induces morphological defects in these organelles. Moreover, addition of oleic acid, which tilts the balance towards elevated levels of MUFA, produces IDH1^*mut*^-specific cellular apoptosis. Collectively, these results suggest that IDH1^*mut*^-induced SCD overexpression can rearrange the distribution of lipids in the organelles of glioma cells, providing new insight into the link between lipid metabolism and organelle morphology in these cells, with potential and unique therapeutic implications.

## Abstract (web summary)

The understanding of altered lipid metabolism by isocitrate dehydrogenase 1 (IDH1) mutations in gliomas at a compartment-specific level is limited. Here, the authors use Raman spectroscopy to monitor organelle-specific metabolic changes and report that IDH1 mutations induce phospholipid imbalances which lead to ER and Golgi dilation.

## Introduction

Altered metabolism has been recognized as a hallmark of cancer cells^1^ and is an area of increased interest for therapeutic targeting. The discovery that mutations in isocitrate dehydrogenase 1 (IDH1) are very prevalent in gliomas^2^, has placed the focus on understanding the metabolome that provides the nutrient milieu favorable for growth and proliferation in this disease. The importance of IDH1 mutations and the associated D-2HG production^3^ to the clinical manifestation of the gliomas is emphasized by the fact that the World Health Organization (WHO) released a novel classification of glioma in 2016 to include IDH1 mutations as molecular markers that dictate the classification^4^ and more recently distinguished grade 4 IDH1^*mut*^ tumors from the IDH1^*WT*^ glioblastoma^5,6^. While major strikes have been made in understanding the effect of IDH1 mutations on glioma biology^2,5,6^, culminating with changes in their classification^4^, the metabolomic-based studies in IDH1^*mut*^ glioma field has been lagging behind. In clinical trials, much effort has been directed toward inhibiting D-2HG formation^7,8^. Although successful in other non-CNS malignancies^9–11^, the clinical benefit of targeting formation of D-2HG in gliomas is still to be determined, creating doubt that this strategy would be effective. Understanding the consequences of IDH1 mutations on cellular function could lead to the discovery of novel therapeutic strategies that are not targeting D-2HG formation directly.

Cytosolic wild-type IDH1 enzyme (IDH1^*WT*^) is involved in the reversible interconversion of isocitrate and αKG utilizing NADP^+^^12^. The tight connection between this reaction and the citrate pool, makes IDH1 a key factor in the regulation of lipid biosynthesis^13^. The loss of the wild-type (WT) allele in gliomas together with acquisition of arginine 132 to histidine mutation leads to impaired citrate formation^12,14^. Moreover, the neomorphic activity of mutant IDH1 utilizes NADPH to synthesize up to 10 mM of D-2HG, thus limiting the NADPH for lipogenesis^15,16^. The combined effect of losing the wild-type allele and usage of NADPH by the mutated one leads to relative depletion of those precursors of lipid biosynthesis. This liability raises the possibility that IDH1^*mut*^ glioma cells have their lipid pools significantly altered. Despite the direct impact of IDH1 mutation on lipid biosynthesis, little is known about how this altered lipid metabolism affects cellular function specifically in IDH1^*mut*^ gliomas.

Lipid metabolism is tightly regulated and highly compartmentalized. While small molecules such those of glycolysis and TCA cycle can diffuse in the cytosol, lipids require chaperon proteins, vesicles, or close contact between membranes for transportation towards their different destination in the cell^17^, thus limiting the interpretation of the data obtained via whole-cell lipidomic approaches. Indeed, classical metabolomic investigations focus on the averaged metabolism of millions of cells and do not reflect fluctuations at the cellular or subcellular level, but organelle or “spatial” metabolomics can detect subcellular abnormalities induced by a disease or treatment. One major challenge in understanding the impact of altered lipid metabolism induced by IDH1^*mut*^ stems in our technical inability to determine and quantify compartment-specific lipidomics^18–23^. Thus, we sought to develop tools for organellar quantification of lipids, which can be applied to determine the lipid profile changes due to IDH1 mutation in organelles of glioma cells.

Recently, we introduced an automated Raman micro-spectroscopy approach, which allows us to quantify and monitor biomolecular composition in single organelles of live cells^24,25^. In the context of lipidomics, this approach facilitates (a) measuring total lipid accumulation, (b) identifying phospholipids and sterols, and (c) partially characterizing the structure of lipids, including the degree of unsaturation and the ratio between cis and trans isoforms.

Here we show via Raman microscopy–based spatial metabolic profiling of live glioma cells that IDH1^*mut*^ overexpression leads to increased heterogeneity in lipid distribution across all organelles and produces an enhanced lipid unsaturation in the ER. Using liquid chromatography (LC)/MS-based organelle lipidomics, we identify higher levels of saturated fatty acids (SFAs) and monounsaturated fatty acids (MUFAs) in the ER, which are subsequently incorporated into phospholipids (phosphatidylethanolamines and phosphatidylcholines) of the ER membrane. To gain insight into the effects of these alterations on organelle function, we perform proteomics analyses, confocal and transmission electron microscopy (TEM). Our analyses reveal unique dilation in ER and Golgi apparatus attributable to the IDH1^*mut*^ and mediated by the D-2HG-induced upregulation of stearyl-CoA desaturase, the rate-limiting enzyme of MUFA biosynthesis. These findings are recapitulated in tumor tissue from patients and point to vulnerabilities that can be exploited therapeutically.

## Results

### IDH1 mutations induce heterogenous lipid composition and increase their degree of unsaturation in organelles

**Fig. 1:**
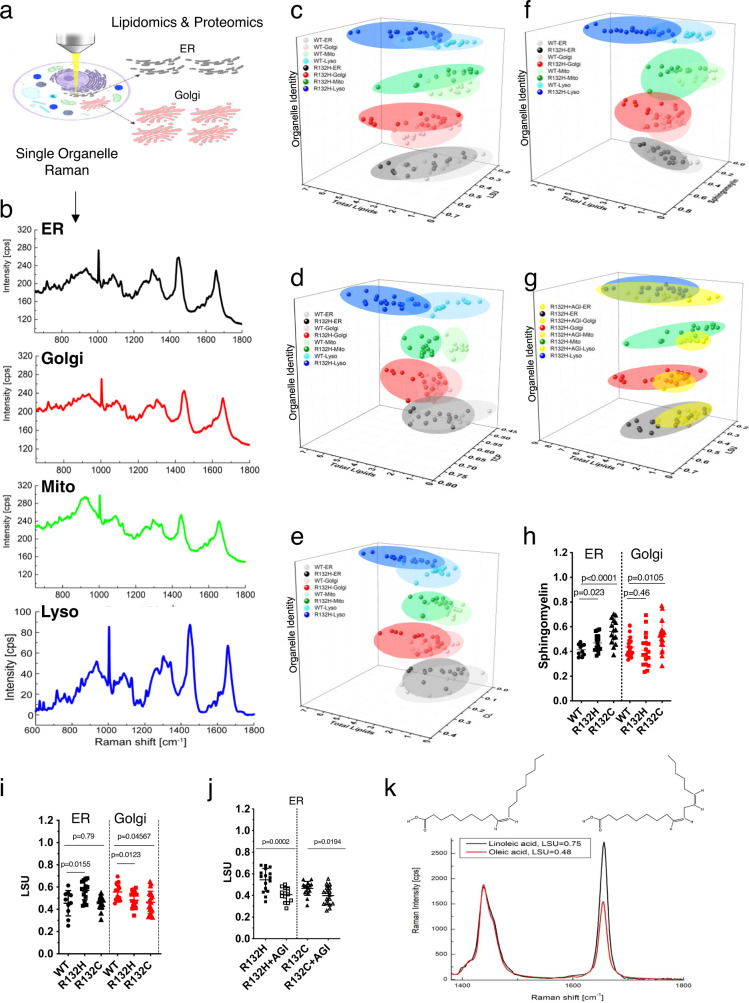
Global biomolecular changes induced by IDH mutation in live cells at the organellar level. **a** Schematic representation of the strategy for the study. Figure was created using Biorender. **b** Representative Raman spectra of live cells obtained using our newly developed method (black, ER; red, Golgi; green, mitochondria; blue, lysosomes). **c**–**f** Distribution of lipid unsaturation parameter, sphingomyelin and TCP parameter in U251^*WT*^ and U251^*R132H*^ cells, represented for each cell and organelle to show the heterogeneity in lipid distribution across cells and organelles. Each point represents the average of three measurements in one organelle of a live cell. Between 10-30 organelles are depicted for the distribution of the parameters per organelle and the data are also^47^ available in Source Data file. Light ovals depict U251^*WT*^ organelle data; dark ovals, the U251^*R132H*^ data. **g** The distribution of the lipid unsaturation parameter becomes more homogeneous after addition of AGI5198, the inhibitor of IDH1 mutation (yellow ovals). Each point represents an average of three measurements in one organelle of a live cell. **h**, **i** Averaged sphingomyelin and LSU levels for ER and Golgi apparatus depicting the changes in this parameter as a function of the mutations. For **h**, sphingomyelin values were determined from the *n* = 15 (U251^R132C^) *n* = 10 (U251^WT^) and *n* = 16 (U251^R132H^) biologically independent samples corresponding to the ER, while from *n* = 21 (U251^R132C^), *n* = 22 (U251^WT^) and *n* = 18 (U251^R132H^) biologically independent samples corresponding to the Golgi apparatus. Data are presented as mean values ± SD. *P* values were determined by a one-way ANOVA followed by Tukey’s test for multiple comparisons for ER and Golgi. For **i**, LSU values were determined from the *n* = 15 (U251^R132C^), *n* = 10 (U251^WT^), and *n* = 14 (U251^R132H^) biologically independent samples corresponding to the ER, while from *n* = 21 (U251^R132C^), *n* = 15 (U251^WT^), and *n* = 17 (U251^R132H^) biologically independent samples corresponding to the Golgi apparatus. Data are presented as mean values ± SD. *P* values were determined via a one-way ANOVA followed by Tukey’s test for multiple comparisons for ER and Golgi. **j** Averaged LSU parameter in ER of both mutants of IDH1 as a function of AGI5198. LSU parameter values were determined from *n* = 16 (U251^*R132H*^), *n* = 14 (U251^*R132H*^ + AGI5198), *n* = 15 (U251^*R132C*^), and *n* = 17 (U251^*R132C*^ + AGI5198) biologically independent samples corresponding to the ER. *p* values were obtained from a two-sided *t*-test with Welch correction for each mutant (R132H and R132C) vs AGI5198 treatment. **k** Raman spectra of pure standards oleic (red) and linoleic acid (black) to show differences in the LSU parameter.

Despite the role of IDH1 enzyme in lipogenesis and its frequent mutation in gliomas, very few studies have addressed the impact of IDH1 mutation to lipid synthesis. Moreover, there was no technology capable of measuring lipids in situ, at the site of their synthesis such as endoplasmic reticulum. To understand the role of IDH1 mutation on lipid metabolism at the organelle level, we recently developed a Raman spectroscopy method to selectively detect and quantify major types of biomolecules in live cells^24,25^ (Fig. 1a). Deconvolution of Raman spectra by *BCAbox* software allowed us to analyze lipid vibrational bonds that belong to different lipid species (Fig. 1b)^24^. The Raman-based findings in live cells were complemented by conventional targeted lipidomic analyses of isolated organelles. We used this approach to profile cells with wild-type IDH1 (IDH1^*WT*^) and cells with the R132H or R132C IDH1 mutations (IDH1^*R132H/C*^) previously described by our collaborators^26,27^, and which generated different concentrations of D-2HG (Supplementary Fig. 1a). Lipid parameters were significantly altered across all organelles compared with the other components of the biomolecular analysis conducted by Raman (Supplementary Fig. 1b). Raman spectral analysis showed that introducing the IDH1 mutation into a U251 glioblastoma cell line (U251^*R132H/C*^) increased lipid heterogeneity, as measured by the following parameters: (1) the lipid unsaturation parameter (the double bond contents, LSU) (Fig. 1c, i, j Supplementary Fig. 2d–f), (2) the trans/cis parameter (stereoisomers of fatty acids, TCP) (Fig. 1d, Supplementary Fig. 2g–i), (3) sphingomyelin (Fig. 1f, h, Supplementary Fig. 1e–g) and cholesterol levels (CL, Fig. 1e, Supplementary Fig. 2a–c). In all organelles except lysosomes, the heterogeneity in distribution of lipid species decreased after addition of AGI5198, a specific inhibitor of the IDH1 mutation (Fig. 1g, yellow ovals, Supplementary Figs. 1e–g and 2). The LSU parameter extracted from intensities at 1440 cm^−1^ and 1660 cm^−1^ of organelle-specific Raman spectra, quantified the degree of unsaturation in lipids (Fig. 1j, Supplementary Figs. 1c and 2d–f)^25^. Interestingly, the average LSU parameter was significantly higher in U251^*R132H*^, but not statistically significant in U251^*R132C*^ compared to U251^*WT*^ in the ER (Fig. 1j). A higher LSU parameter suggested more lipids with C = C bonds accumulated at U251^*R132H/C*^ cells based upon external calibration with oleic and linoleic acid (Fig. 1k). By contrast, the LSU parameter was decreased in Golgi apparatus of both mutant cells. Since the ER is the site of lipid synthesis and Golgi apparatus is responsible for their modification and sorting, we further analyzed their lipid composition through LCMS analysis of extracted organelles from IDH1^*mut*^ and IDH1^*WT*^ cells.

### IDH1 mutations lead to accumulation of SFA-, and MUFA-PEs and PCs in the ER and their depletion from Golgi apparatus

**Fig. 2:**
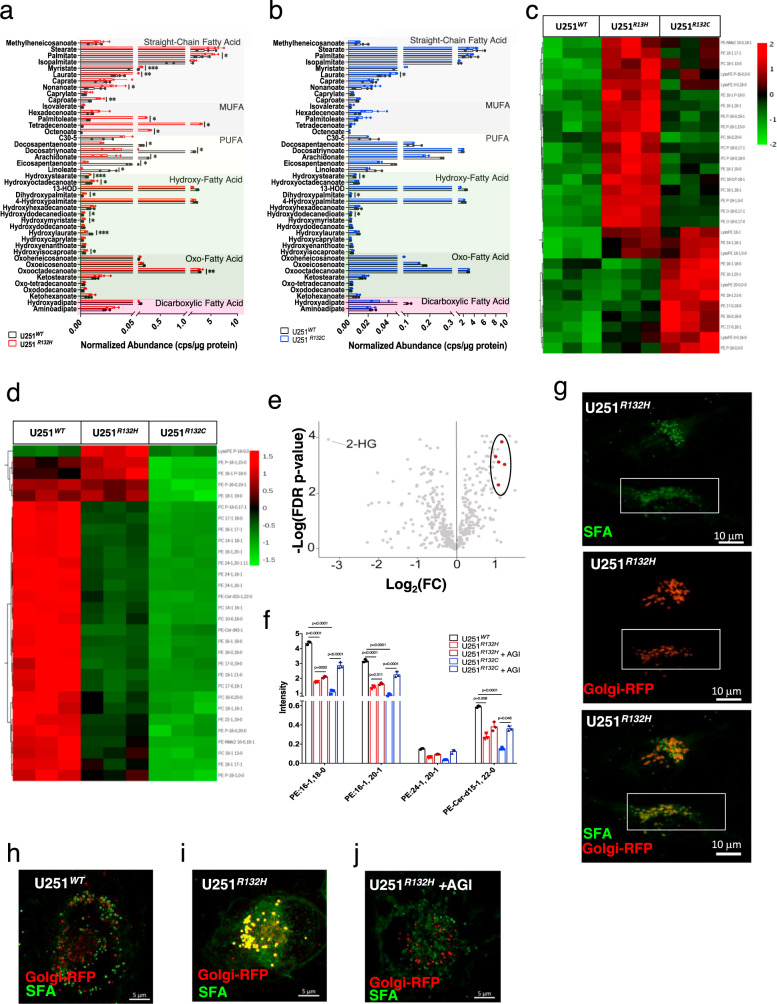
Endoplasmic reticulum (ER) and Golgi apparatus-specific lipid changes due to IDH1 mutation. Comparison of the lipidomic profiles of ER from U251^*WT*^ (black) and U251^*R132H*^ (red) (**a**) or U251^R132C^ (blue) (**b**) revealed a higher relative abundance of saturated and monounsaturated fatty acids in U251^R132H^ cells only, similar to the LSU parameter. Black bars represent values for U251^*WT*^ cells (*n* = 3) while the red and blue represent U251^*R132H*^ (*n* = 3) and U251^*R132C*^ (*n* = 3), respectively. The mean values and standard deviation (error bars) are represented. Statistical values are represented as: ns not significant; *p ≤ 0.05; **p ≤ 0.005; ***p ≤ 0.0005; ****p ≤ 0.0001 and were determined using two-sided student *t*-test with Welch correction. **c** Heatmap of phospholipids extracted from the ER of U251^*WT*^, U251^*R132H*^, and U251^*R132C*^ cells shows the 30 most significantly altered features. **d** Heatmap with the most significant phospholipids from the targeted Golgi lipidomics showed that most phospholipids containing saturated or monounsaturated phospholipids are depleted in Golgi. Heatmaps were created using MetaboAnalyst. **e** Volcano plot of the lipidomic assay comparing U251^*WT*^ and U251^*R132H/C*^ combined. 2HG appears to be the most significant metabolite upregulated in the mutant cells, whereas phospholipids (PEs) (red dots) are among the most downregulated lipids in mutant cells. *P* values were obtained via two-sided Student’s *t*-test followed by FDR correction. **f** Relative intensity of PEs that contain zero or one double bond are downregulated in mutant cells (light red and light blue bars) compared with wild-type (black bars) and are partially restored by adding AGI5198 inhibitor (dark red and dark blue bars). Mean values and SD are represented. Error bars represent standard deviation of *n* = 3 independent experiments. One-way ANOVA test with Tukey’s correction for multiple comparisons was conducted using GraphPad Prism 8.2.1. **g** Z-stacked Golgi reconstructed image from fluorescence microscopy shows the area of colocalization between Golgi apparatus (red) and saturated fatty acids (green). Scale is 10 microns. Images were collected using fast frame switching in lattice SIM mode with 0.1 um z-step size, 0.03 um X–Y pixel size, and processed using the SIM module of the Zen software Images are representative of *n* = 3, independent experiments. **h**–**j** Fluorescence microscopy shows colocalization (yellow, middle panel) of saturated fatty acids (green) with the Golgi apparatus (red) in U251^*R132H*^ cells (**i**) and the loss of colocalization in the presence of the inhibitor AGI5198 (**j**). Scale is 5 microns. At least five images per group were taken for each experiment. Images are representative of *n* = 3, independent experiments.

Since the LSU parameter obtained from Raman only measured the amount of unsaturation, we employed a targeted lipidomic assay via LC/MS of isolated ER and Golgi in order to identify and quantify the different MUFA-containing lipid species altered. U251^*R132H/C*^ glioma cells showed a higher relative abundance of SFAs and MUFAs within the ER and a lower relative abundance of polyunsaturated fatty acids (PUFAs) compared to IDH1^*WT*^ cells (Fig. 2a, b). The abundance of SFAs and MUFAs incorporated into the phospholipids from ER in both mutant cells varies at different degrees, as shown in the heatmap (Fig. 2c). Our method is able to detect and quantify the nonpolar lipidome comprised of fatty acids, acylcarnitines, fatty acid primary amide, eicosanoids, prostaglandins, amino-acyl endocannabinoids, triglycerides, ceramides, glycoceramides, sulfatide as well as numerous classes of glycerophospholipids—PE, LPE, PC, LPC,,PS, LPS, PG, PIP (Supplementary Fig. 3a). Amongst the classes of phospholipids with MUFA, phosphatidylethanolamine (PE)-MUFAs were the most prevalent to be altered followed by MUFA-containing phosphatidylcholines (PCs). We also detected few MUFAs incorporated into Lysophosphatidylethanolamines (LPE), PEs, phosphorylated PEs (PE-P), and PE-Ceramides (PE-Cer).

Addition of 12.5 μM AGI5198, a specific inhibitor of D-2HG production, specifically reversed the levels of phospholipids in these variants of IDH1, suggesting a correlation between D-2HG levels and this phospholipid imbalance (Supplementary Fig. 3a, b). The impact of IDH1 mutation on the increased relative abundance of SFA and MUFAs was more pronounced in the U251^*R132H*^ than in U251^*R132C*^ cells, this difference may be attributed to higher D-2HG levels produced by the R231H mutant (Supplementary Fig. 1a). This data were consistent with higher average LSU parameter observed in ER of U251^*R132H*^ compared with U251^*R132C*^ cells (Fig. 1i) and the LSU decrease in presence of AGI5198. Together, these results show that phospholipid imbalance in ER and Golgi is correlated with the levels of D-2HG.

Our analyses also revealed downregulation of SFA and MUFA-phospholipids in Golgi apparatus of these cells as a result of both IDH1 mutations (Fig. 2d). Indeed, the most downregulated features, as seen in the heatmap (Fig. 2d) or the volcano and bar plots (Fig. 2e, f) when U251^*R132H/C*^ was compared with U251^*WT*^, were the SFA- and MUFA-based PEs and PCs, which were partially restored by adding AGI5198 (Fig. 2f). To confirm the deregulation of PEs and PCs at the organelle level due to IDH1 mutation in live cells, we stained the ER, Golgi, mitochondria and lysosomes with red fluorescence protein (RFP)-proteins and added BODIPY^TM^ FL C16 (4,4-Difluoro-5,7-Dimethyl-4-Bora-3a,4a-Diaza-s-Indacene-3-Hexadecanoic Acid) to visualize the uptake and tracing of this SFA’s fate by using confocal microscopy. Although BODIPY-palmitate appears to be ubiquitously distributed throughout the cytoplasm, we observed no uptake of BODIPY-palmitate in the ER, mitochondria or the lysosomes by the lack of colocalization with the resident membrane red fluorescent protein in U251^*R132H/C*^ or U251^*WT*^ cells (Supplementary Fig. 3c–e). Prominent colocalization of BODIPY-palmitate with the Golgi-resident protein N-acetylgalactosaminyltransferase was observed specifically in U251^*R132H*^ and not in U251^*WT*^cells (Fig. 2g, h); this effect could be reversed by adding AGI5198 (Fig. 2i, j). Specific uptake of SFA by Golgi organelles indirectly suggests the lack of SFA in Golgi and correlates with the depleted PEs observed via MS-based lipidomics.

### Mutant IDH1-induced SCD expression and phospholipid imbalance lead to ER and Golgi dilation

**Fig. 3:**
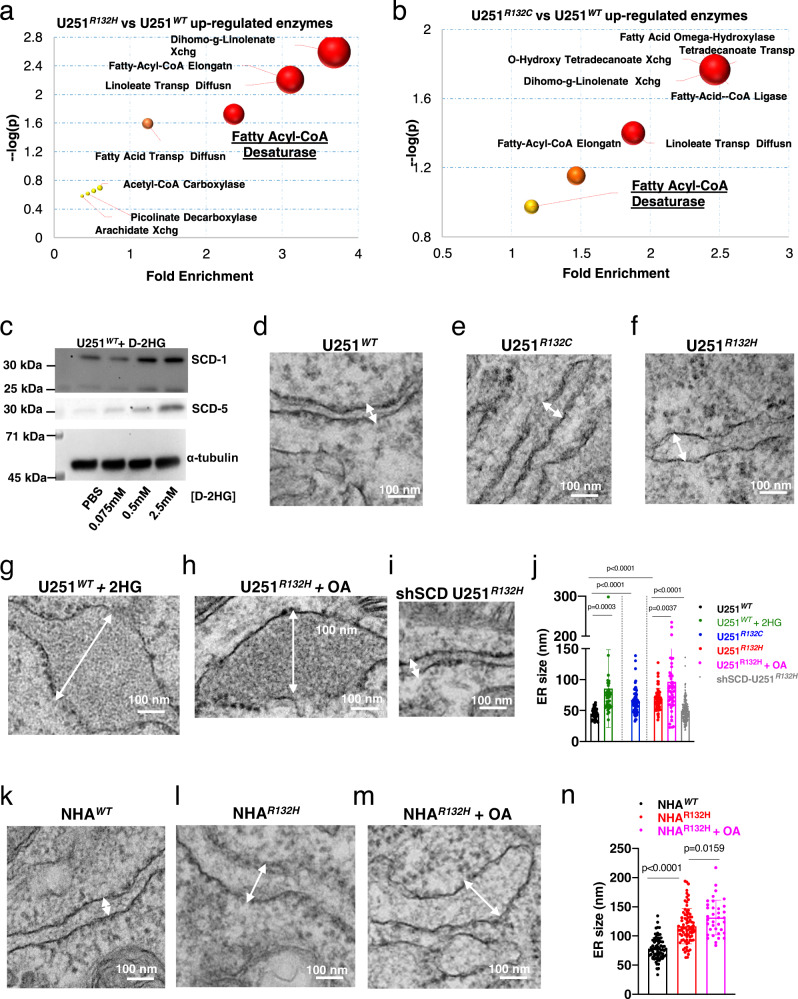
Stearyl CoA desaturase (SCD) overexpression is induced by D-2HG and is responsible for IDH1^*mut*^-induced defects in ER. **a,** **b** Predicted enzymes from Golgi-specific lipids identified from U251^*R132H/C*^ by mass spectrometry. Ratios of lipid levels between U251^*R132H/C*^ and U251^*WT*^ were used in MetaboAnalyst^47^ to predict enzymes most affected. **c** Western blot analysis show SCD-1/5 expression upon addition of 0.5–2.5 mM D-2HG to U251^*WT*^. Uncropped blots are available in Source data. Experiments were performed in duplicates (SCD-5) or triplicates (SCD-1). **d**–**f** Transmission electron micrographs of U251^*WT*^, U251^*R132H*^, and U251^*R132C*^ show significant changes in the ER structure. Arrows indicate the distance between two ER membranes. **g** TEM micrograph showing high resolution images of ER U251^*WT*^ upon addition of D-2HG, which leads to ER dilation. **h** TEM micrograph showing high resolution images of ER U251^*R132H*^ upon addition of oleic acid, which further leads to ER dilation. **i** ER morphology in U251^*R132H*^ cells recovered upon knocking down of *hSCD* via short hairpin RNA. **j** Quantification of ER dilation was done via measurements of the distance between the two ER membrane. U251^WT^ (black symbols) (*n* = 39), U251^*R132C*^ (*n* = 69, blue symbols), U251^*R132H*^ (*n* = 66, red symbols) U251^*WT*^ + D-2HG (*n* = 39, green symbols), U251^*R132H*^ + oleic acid (OA, *n* = 51, magenta symbols), U251^*R132H*^ + *shSCD* (*n* = 198, gray symbols). **k**–**m** TEM micrograph showing high resolution images of ER in NHA^*WT*^, NHA^*R132H*^, and NHA^*R132H*^ treated with oleic acid cells. **n**. Quantification of ER lumen enlargement via measurements of the distance between the two ER membrane in normal *human* astrocytes (NHA). NHA^*WT*^ (black symbols) (*n* = 79), NHA^*R132H*^ (*n* = 80, red symbols), NHA^*R132H*^ + oleic acid (OA, *n* = 36, magenta symbols). For **j** and **n** the mean values ± SD are presented. *p* values from a one-way ANOVA followed by Tukey’s test for multiple comparisons were obtained using GraphPad Prism 8.2.1 for comparison of each group. Data are available in Source Data file. At least 10 images per organelles were taken. Micrographs are representative of three independent experiments.

To understand the link between the IDH1 mutation and the imbalance in lipid distribution we conducted enzyme enrichment analysis using MetaboAnalyst^28,29^ and the levels of metabolites extracted from Golgi. We found that desaturases, hydrolases, and lipid transport were the major upregulated enzymes in the mutated cells (Fig. 3a, b). Enzyme prediction also showed that fatty acid desaturases in U251^*R132H*^ cells were more significantly upregulated from the metabolite trends than did those in U251^*R132C*^ cells. Since stearyl-CoA desaturases are the key regulators of lipid homeostasis, by catalyzing the rate-limiting step in MUFA biosynthesis in ER^30^ we set out to investigate the link between *human* SCD (hSCD) enzymes and the lipid imbalances associated to IDH1 mutation. Two isoforms of SCD enzymes are present in *humans*: SCD-1 and SCD-5. We treated U251 IDH1^WT^ cells with D-2HG and measured the expression of both hSCD enzymes. hSCD enzymes’ expression was augmented with increasing concentrations of D-2HG in a dose-dependent fashion (Fig. 3c). Thus, the upregulation of SCD-1 and 5 enzymes provides the link between IDH1 mutations and the imbalance in phospholipids observed in ER and Golgi.

Given the importance of phospholipids in membrane integrity, we next explored the link between the organelle’s morphology and phospholipid imbalance in the ER and Golgi by Transmission Electron Microscopy (TEM). We measured the ER size from images captured by TEM of U251 glioblastoma and normal human astrocyte (NHA) cells with or without IDH1^*R132H*^ mutation. Surprisingly, ER swelling was frequently detected in the IDH1^*R132H*^ mutant cells (Fig. 3d–f). The mean distance between the two ER membranes increased from 45 ± 1.5 nm in the U251^*WT*^ cells to 65 ± 2.4 nm for the U251^*R132C*^ and 69 ± 2.2 nm for U251^*R132H*^ cells, respectively. We then investigated whether ER morphology could be directly altered by D-2HG. Interestingly, addition of 5 mM D-2HG to U251^*WT*^ cells led to a 1.9-fold increase in the mean length of the two ER membranes (85 ± 10 nm) of U251^*WT*^ cells, with certain ER areas measuring over 100 nm in distance between the two membranes (Fig. 3g, j, green bars). IDH1 mutation induced ER dilation in a second model system, obtained by overexpression of either IDH1^*WT*^ or IDH1^*R132H*^ in NHA^31^ (Fig. 3k, l, n). Overall, these results indicate that IDH1 mutations are inducing ER dilation via D-2HG production.

To probe the hypothesis that increasing MUFA levels can further induce ER dilation, we added oleic acid, a prominent MUFA produced by hSCD enzymes to either U251^*R132H*^ or NHA^*R132H*^ cells. Addition of oleic acid to either U251^*R132H*^ of NHA^*R132H*^ cells led to further dilation of ER membranes with regions that measured over one hundred nanometers in size (Fig. 3h, j, magenta bars, Fig. 3m, n, magenta bars). Having demonstrated a significant upregulation of hSCD enzymes, we next knockdown this enzyme via short hairpin RNA in U251^*R132H*^ cells to test the hypothesis that lowering MUFA levels in ER will restore its morphology. Indeed, we found that this genetic alteration was sufficient to restore ER size close to U251^*WT*^ (Fig. 3i, j, gray bars). There results collectively, point to a model in which IDH1-induced hSCD expression increased MUFA levels and ER dilation.

**Fig. 4:**
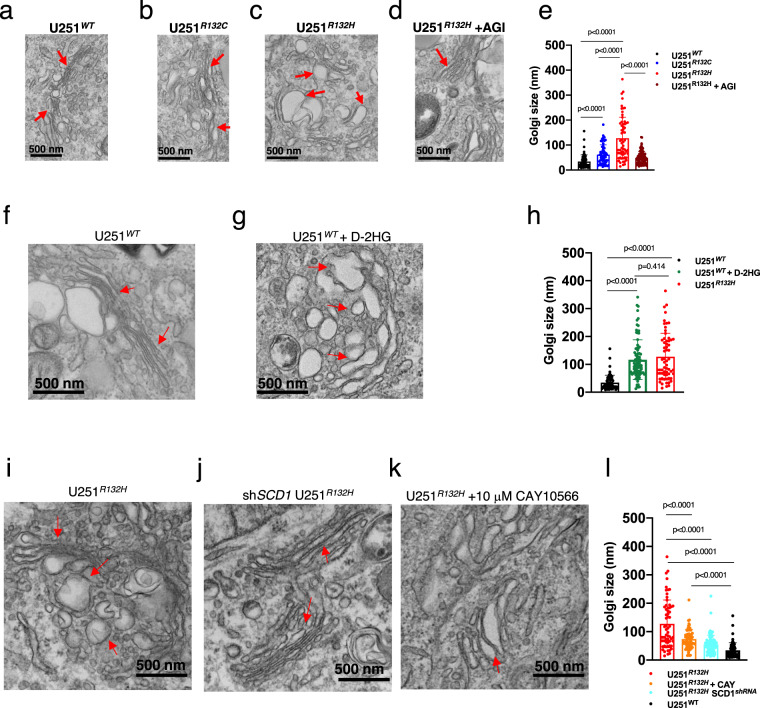
D-2HG levels and SCD expression are responsible for IDH1^*mut*^-induced membrane defects in Golgi. **a**–**d** Transmission electron micrographs of U251^*WT*^, U251^*R132C*^ U251^*R132H*^, and U251^*R132H*^ + AGI5198 show significant changes in the Golgi structure. Arrows indicate the altered region of Golgi stacks. **e** Golgi size quantification of U251^*WT*^ (*n* = 63, black symbols), U251^*R132C*^ (*n* = 59, blue symbols), U251^*R132H*^ (*n* = 63, red symbols), and U251^*R132H*^ + AGI5198 (*n* = 90, dark red symbols). Statistical values are represented on the graph. **f**, **g** Addition of D-2HG in U251^*WT*^ was enough to cause Golgi dilation. **h** Golgi size quantification of U251^*WT*^ (*n* = 63, black symbols), U251^*WT*^ + D-2HG (*n* = 90, green symbols), and U251^*R132H*^ (*n* = 63, red symbols). **i**–**j** Comparison between Golgi of U251^*R132H*^ cells and the U251^*R132H*^ cells that lack hSCD showed a restored Golgi structure. **k** Inhibiting the hSCD enzyme with CAY10566 led to partial restoration of Golgi structure. **l** Golgi size quantification of U251^*R132H*^ (*n* = 63, red symbols), U251^*R132H*^ + CAY10566 (*n* = 69, orange symbols), and siRNA hSCD U251^*R132H*^ (*n* = 84, cyan symbols). For **e**, **h**, **l** the mean values and SD are presented and the *p* values obtained from a one-way ANOVA tests with Tukey’s correction for multiple comparisons were conducted using GraphPad Prism 8.2.1. At least 10 images per organelles were taken. Data are available in Source Data file. Micrographs are representative of three independent experiments.

Moreover, TEM studies showed that Golgi morphology was even more profoundly altered in mutant cells compared with the ER. Golgi cisternae were significantly enlarged and swollen in IDH1^*R132H*^ cells (average Golgi size of 126 ± 10 nm) and to a lesser extent in IDH1^*R132C*^ cells (average Golgi size of 62 ± 5.1 nm) compared with IDH1^*WT*^ cells (34 ± 3.4 nm) (Fig. 4a–c, e). Having demonstrated that decreasing the concentration of D-2HG via AGI5198 in U251^*R132H*^ cells restored phospholipid levels in Golgi (Fig. 2f), we also probed whether this inhibitor restored the Golgi size and morphology. Indeed, the average Golgi size was decreased from 126 ± 10 nm to 49 ± 2.7 nm upon addition of AGI5198 inhibitor in U251^*R132H*^ cells (Fig. 4e, dark red bars). Since U251^*R132H*^ and U251^*R132C*^ cells differ in the level of D-2HG that they produce (Supplementary Fig. 1a), we next tested whether addition of D-2HG levels to U251^*WT*^ cells is sufficient to induce the dilated Golgi phenotype observed in U251^*R132H*^ cells. Similar to effect of D-2HG on the ER morphology addition of 5 mM D-2HG to U251^*WT*^ cells induced that dilated phenotype by increasing the Golgi size significantly (117 ± 7.5 nm) compared with the nontreated cells (34 ± 3.4 nm) (Fig. 4h). Interestingly, we found no significant difference between the mean Golgi size of U251^*R132H*^ (126 ± 10 nm) and U251^*WT*^ + D-2HG (117 ± 7.5 nm) cells suggesting that D-2HG mimics the effects of R132H mutation on this organelle morphology (Fig. 4h). Since hSCDs are central lipogenic enzymes for the synthesis of MUFA^32^ and were found to be upregulated by D-2HG levels, we next probed the effect of downregulating one of this enzymes on Golgi morphology. Similar to the ER, knockdown of hSCD via short hairpin RNA restored the Golgi structure and size in U251^*R132H*^ cells (55 ± 3.5 nm), as measured by TEM and quantified by the size measurements (Fig. 4j, l). However, inhibition of hSCDs activity via addition of Cay10566, only partially restored the degree of Golgi dilation (74 ± 4.0 nm); therefore, a more pronounced recovery of Golgi cisternae was obtained by decreasing hSCD expression as compared with hSCD inhibition (Fig. 4k, l). Together these results support a working model in which D-2HG induces hSCDs overexpression and Golgi dilation.

### SCD overexpression, ER and Golgi dilation are prevalent in tumor samples of oligodendroglioma, but not in GBM

**Fig. 5:**
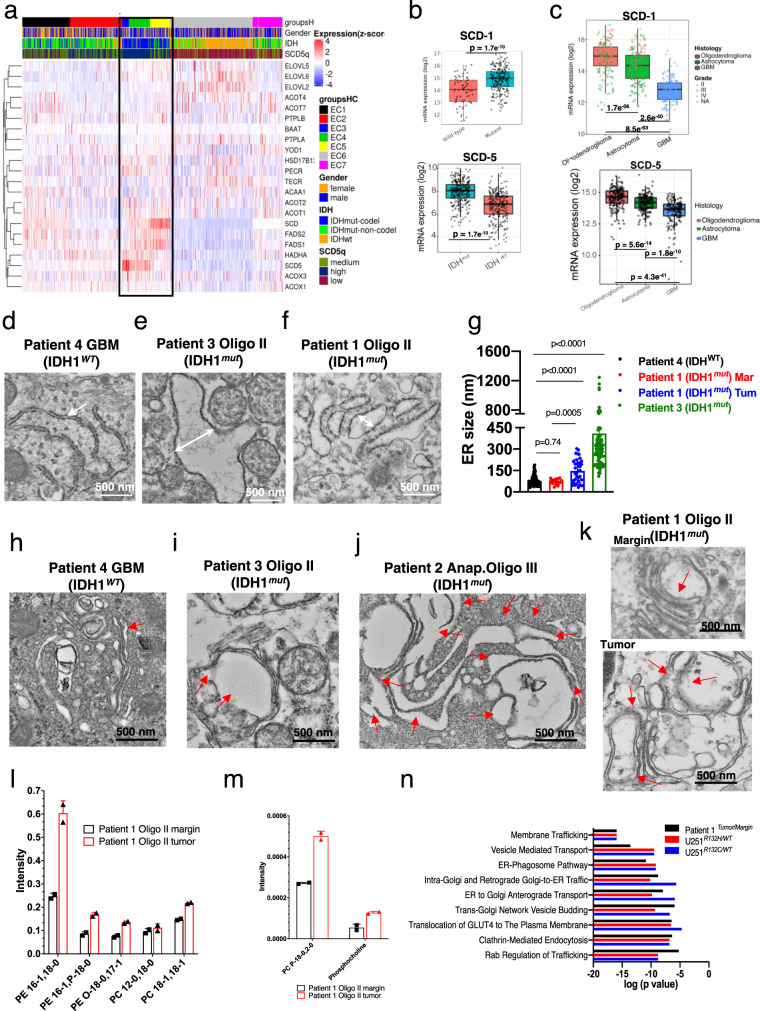
Validation of SCD expression, phospholipid imbalance, and Golgi dilation in patient tissue and their impact on cellular function. **a** Consensus clustering of 22 genes from fatty acid synthesis pathway reveled clustering of oligodendroglioma samples with highest mRNA levels for SCD-1 and SCD-5 enzymes. **b** mRNA levels of SCD-1 and SCD-5 from The Cancer Genome Atlas show increased expression in these transcripts in IDH1^*mut*^ (*n* = 218) tissue compared with IDH1^*WT*^ (*n* = 68) in low-grade gliomas (LGG). **c** mRNA levels of SCD-1 and SCD-5 were inversely correlated with the molecular subtypes in the following order: oligodendroglioma (*n* = 161), astrocytoma (*n* = 194), and GBM (*n* = 152). Figure was created using GlioVis data portal^51^. Box plots indicate median (middle line), 25th, 75th, percentiles (box), and 5th and 95th percentile (whiskers) as well as outliers. For **b** and **c** pairwise *t*-test comparisons between group levels with Bonferroni correction was used to determine the *p* value shown on the plot. **d**–**f** TEM micrograph showing high resolution images of ER for tissue from three patient samples; two oligodendroglioma and one glioblastoma multiforme. At least 10 images per organelles were taken. Micrographs are representative of three independent experiments. **g** Distance measurements of ER membranes from tissues of: Patient 4 (GBM, black symbols, *n* = 106), Patient 1 margin (red symbols, *n* = 17), Patient 1 tumor (blue symbols, *n* = 37), and Patient 3 (green symbols, *n* = 100). One-way ANOVA followed by Tukey’s test for multiple comparisons was conducted using GraphPad Prism 8.2.1. Data are plotted as mean ± SD. **h**–**k** Transmission electron micrographs of Golgi in tissue from different grades of oligodendroglioma compared with glioblastoma multiforme (GBM) (IDH1^*WT*^) show specific dilation of this organelle (red arrows) in the oligo tumor samples only. At least 10 images per organelles were taken. Micrographs are representative of three independent experiments. **l**, **m** Increased MUFA- phospholipids in the Golgi of a tumor from patient 1 compared with the margin. Data are shown as mean ± SEM and are representative of two technical replicates. **n** Reactome pathways for Golgi proteins extracted from tissue of Patient 1 (black) U251^*R132H/WT*^ (red), U251^*R132C/WT*^ (blue). The tables are describing the statistics for the top three pathways altered based upon their *p* value for the three comparisons. *P* values were corrected for the multiple testing (Benjamini–Hochberg procedure) that arises from evaluating the submitted list of identifiers against every pathway. Note that both variants were analyzed separately; however, their results were similar for the top three pathways. The graphs depict the significance of the specific pathways under the membrane trafficking with the corresponding p values for three comparisons Patient 1 (black bars), U251^*R132H/WT*^ (red bars*)*, U251^*R132C/WT*^ (blue bars). Data were plotted using Reactome-based analysis report tables, which are located in Supplementary Tables 2–4.

To determine the prevalence of hSCDs overexpression across all gliomas subtypes and throughout the fatty acid biosynthetic pathway, we used RNA-seq data from The Cancer Genome Atlas (TCGA) and compared the mRNA levels of twenty-two fatty acid biosynthesis genes in IDH1^*mut*^ and IDH1^*WT*^ tissue. Consensus clustering of mRNA for all these fatty acid synthesis genes revealed a striking high expression of hSCDs in oligodendroglioma tissues (Fig. 5a). Consistently, the levels of hSCDs-mRNA in IDH1^*mut*^ lower grade gliomas were significantly higher than those in IDH1^*WT*^ tumors (Fig. 5b). We also compared mRNA expression across all histological types and grades of gliomas and obtained similar results; patients with oligodendroglioma had the highest levels of hSCDs-mRNA, followed by those with astrocytoma and lastly GBM (Fig. 5c).

To understand the clinical relevance of our in vitro findings, we acquired fresh tissue from patients with either IDH1^*WT*^ GBM or oligodendroglioma (IDH1^*mut*^). We selected oligodendrogliomas because these tumors presented the highest expression of hSCDs enzymes amongst all glioma subtypes in the consensus clustering analysis above. The patient tissue analyzed in this study had the following characteristics: patients 1 and 2 only had surgery at the time of tissue analysis, whereas all other patients had the standard of care, which included radiation and chemotherapy. Tissue from patients 3 and 5 was from a recurrent tumor, and the others were from primary tumors (Supplementary Fig. 4a). Interestingly, tissue samples from oligodendroglioma tissues (IDH1^*mut*^), although in different stages of disease (Supplementary Fig. 4a), displayed vastly dilated rough ER and Golgi (Fig. 5e–g, i–k), which was not evident in GBM tissue samples (Fig. 5d, g, black symbols, 5 h). Thus, TEM results from three IDH1^*mut*^ (oligodendroglioma) tissue samples and two IDH1^*WT*^ (glioblastoma) tissue samples revealed that IDH1^*mut*^ tumors had high prevalence of ER and Golgi dilation as those found in vitro, while this effect was not present in the IDH1^*WT*^ samples. Since GBM (IDH1^*WT*^) and oligodendrogliomas (IDH1^*mut*^) are very different diseases, we then compared the tumor from the Patient 1 (oligo IDH1^*mut*^) with its margin. Interestingly, Golgi apparatus had normal morphology at the tumor margin, while dilated and swollen Golgi were found in the tumor sample of this patient (Fig. 5k). Mass spectrometry-based lipidomics showed that the tissues from oligodendrogliomas had high levels of MUFAs and PE-MUFAs compared with the margin (Fig. 5l, m, Supplementary Fig. 4b). These results show that expression of hSCDs present the highest levels in oligodendrogliomas with IDH1 mutation amongst all the glioma subtypes, the imbalance in phospholipids is recapitulated in tumors of oligodendroglioma and correlates with ER and Golgi distorted morphology.

### ER-Golgi trafficking pathways are significantly altered in IDH1^*mut*^ tumors and glioma cells

To gain more insight into the implication of this lipid profile on the cellular function and to validate our results, we conducted organelle-specific proteomics. Over 5000 proteins with differential expression between U251^*R132H/C*^ and U251^*WT*^ Golgi cells or tumor versus margin from Patient 1 tissue were uploaded to the Reactome database to create pathway over-representation maps for each comparison^33,34^. All three comparisons yielded the following pathways with the highest score: membrane trafficking, translation and either metabolism of RNA (cells) or axon guidance (tissue) (Supplementary Fig. 4c). Membrane trafficking was one of the most altered pathways amongst all three comparisons with more than 400 proteins differentially expressed (Supplementary Tables 1–3). We expanded this node and looked at the overexpressed subpathways as a function of their *p* value in all of the comparisons (Fig. 5n). Interestingly, in all three comparisons, significant subpathways highlighted ER-Golgi interactions such as: retrograde Golgi to ER, anterograde ER-Golgi transport as well as cellular processes of ER and Golgi independent of each other. Trans-Golgi vesicle budding scored highly together with vesicle mediated transport, Clathrin-mediated endocytosis and Rab regulation of trafficking. All these alterations highlight the upregulation of the secretory membrane system of IDH1^*R132H*^ gliomas, where ER, Golgi complex, plasma membrane, and other vesicles are working together to deliver lipids, newly synthesized proteins, and carbohydrates needed for cell growth^35^.

### IDH1^*mut*^ cell lines are more sensitive to MUFA-induced apoptosis compared with IDH1^*WT*^

**Fig. 6:**
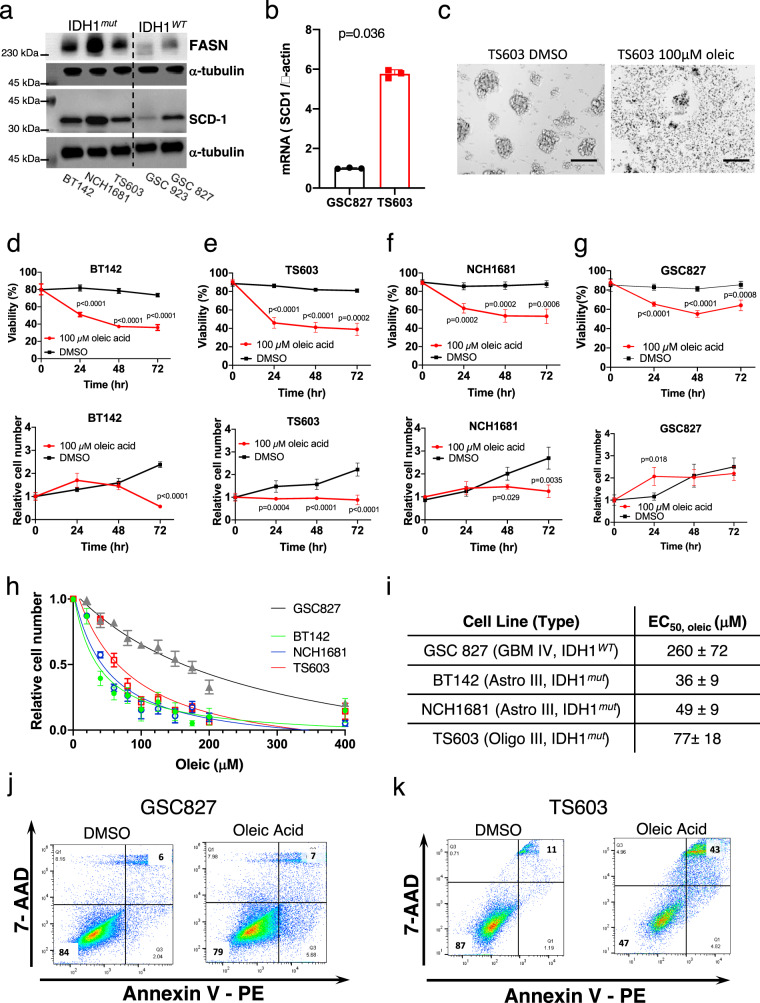
Addition of MUFA leads to IDH1^*mut*^-specific cell death. **a** Western blot analysis of fatty acid synthesis and regulation proteins in a panel of patient-derived cell lines. Uncropped blots are available in Source Data. Experiments were conducted in triplicates. **b** mRNA levels of SCD-1 are is higher in TS603 (IDH1^*R132H*^) compared with GSC827 (IDH1^*WT*^) as determined by RT-PCR. The data have been obtained from three biologically independent samples and is plotted as mean ± SD. *P* = 0.036 was obtained using two-sided Student’s *t*-test with Welch’s correction. **c** Photomicrographs of TS603 neurospheres in absence and presence of oleic acid showing the cell death. Scale bar corresponds to 100 microns. At least five images were taken for each group. **d**–**g** Oleic acid treatment led to decreased viability in patient-derived cell lines BT142, TS603, and NCH1681, and it was more pronounced than in IDH^*WT*^ (GSC827). The average value of five biological replicates is shown along with the standard deviation. *p* values were determined via a two-way ANOVA for multiple comparisons followed by Sidak correction for alpha = 0.05. **h**, **i** EC_*50*_ measurements of oleic acid. from CCK-8 assays, which show greater sensitivity of IDH1^*mut*^ cells (green, blue, and red lines) to oleic acid supplementation compared with IDH1^*WT*^ neutrospheres (gray line). The average value of four (GSC827, NCH1681) or five (BT142 and TS603) biological replicates is shown along with the standard deviation. **j**, **k** In IDH1^*WT*^ cells (GSC827), there was no change in cellular apoptosis measured 48 h after addition of oleic acid. In patient-derived IDH1^*mut*^ cells (TS603) 43% of cells underwent late apoptosis as measured by FITC Annexin V Apoptosis Detection Kit.

hSCDs enzymes are membrane proteins of the ER, and as the rate-limiting enzymes in the biosynthesis of MUFA, they yield two common products: oleyl- and palmitoyl-CoA. Since oleic and palmitoleic acids are the major MUFAs in fat depots and membrane phospholipids, we explored the therapeutic consequences of tilting the balance toward higher levels of MUFAs in IDH1^*R132H*^ cells, that were derived from patients or engineered to overexpress this mutation. Using a panel of IDH1^*R132H*^ patient-derived cell lines from astrocytoma (BT142, NCH1681), as well as oligodendroglioma (TS603), for which we showed synthesis of D-2HG, especially TS603 oligo cell line, as previously reported^36^ (Supplementary Fig. 5a). Moreover, wild-type cell lines of GBM (GSC923, GSC827) were also utilized as controls. We confirmed that SCD-1 enzyme is highly expressed in patient-derived IDH1^*mut*^ cells compared to wild-types, whereas basal expression of SCD-1 was observed in IDH1^*WT*^ patient-derived neurospheres (Fig. 6a, b). Interestingly, fatty acid synthase (FASN) expression followed the same trend as hSCDs when comparing IDH1^*WT*^ and IDH1^*R132H*^ cell lines (Fig. 6a). With this panel, we next explored whether we could manipulate the specific vulnerability induced by IDH1 mutation for therapeutic purposes. Since the most prevalent MUFAs in cells are palmitoleic and oleic acid, we added oleic acid to U251^*R132H/C*^ and the patient-derived IDH1^*mut*^ and IDH1^*WT*^ cell lines described above. Cell proliferation and viability decreased significantly within the first 24 h after treatment with 100 μM oleic acid, as revealed by trypan blue viability assays (Fig. 6c–f, Supplementary Fig. 5b). The same concentration of oleic acid in IDH1^*WT*^ cell lines did not alter their proliferation rate or viability as drastically as it did for IDH1^*mut*^ cells (Fig. 6g). IDH1^*mut*^ cells were more sensitive to oleic acid–induced apoptosis, as measured by EC_50_ using a cell viability kit (CCK-8 assay) and Annexin V and a 7-AAD flow cytometry–based assay. All patient-derived IDH1^*mut*^ cell lines irrespective of their molecular type (oligodendroglioma or astrocytoma) were 4-fold to 7-fold more sensitive to oleic acid–induced cell death, respectively, as measured by EC_50_, compared with WT (GSC827) cells (Fig. 6h, i). Addition of oleic acid increased the percentage of cells that underwent late apoptosis, as observed via flow cytometry (43% apoptotic cells in IDH1^*mut*^ compared with 7% in IDH1^*WT*^) (Fig. 6j, k, Supplementary Fig. 5c). In addition, TEM micrographs showed ER dilation after oleic acid addition (Fig. 3h, j, m, n). Oleic acid changed the morphology of the cells: vesicles resembling lipid droplets appeared inside the cells (Supplementary Fig. 5d), which was confirmed through oil red and neutral lipid staining (LipidTOX Neutral Green, ThermoFisher) (Supplementary Fig. 5g). Inhibition of fatty acid synthase (FASN) or addition of PUFAs (linoleic acid) had a more pronounced effect on U251^*WT*^ cells but did not affect U251^*R132H/C*^ cells significantly, suggesting that the specific vulnerability of IDH1^*mut*^ is in the accumulation of MUFAs and SFA-to-MUFA conversion (Supplementary Fig. 5e, f).

## Discussion

**Fig. 7:**
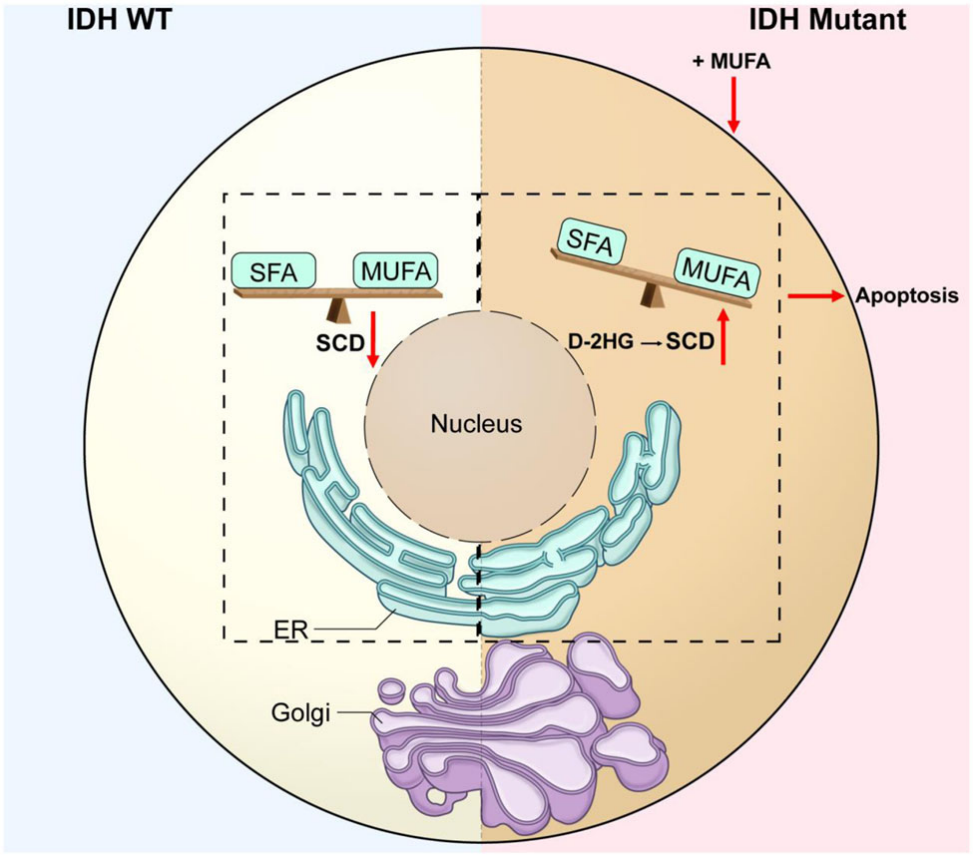
Proposed model for IDH1^mut^-induced phospholipid imbalance and cellular function. IDH1^*WT*^ cells display homeostatic levels of phospholipids and basal expression of hSCD enzymes. IDH1^*mut*^ cells induce high levels of hSCD enzymes, which alter the phospholipid balance and the morphology of ER and Golgi. Shown with red arrows is a potential way to exploit this alteration, by addition of oleic acid a prevalent MUFA, which leads to apoptosis. Dashed rectangle signifies the fact that hSCD enzymes are localized within the ER membrane.

Determining the reprogramming of lipid metabolism in IDH1^*mut*^ glioma, at the site of their synthesis, has been challenging in part due to technical limitations in detecting and quantifying lipids with such accuracy^37^. These technical challenges have prevented a deeper understanding of the role of tumor-induced metabolic alterations on cellular function. Herein, applying our recently developed Raman spectroscopy-based biomolecular profiling of organelles^24,25^, we identified a lipid imbalance in ER and Golgi in the context of IDH1^*mut*^ gliomas, that was previously unknown. We validated our findings from Raman spectroscopy with organelle lipidomics, proteomics and morphological characterization of these two central organelles involved in lipid synthesis and trafficking. We found that stearyl-CoA desaturase was significantly upregulated in cells by D-2HG and had the highest mRNA levels in tissues from oligodendrogliomas. Importantly, we demonstrated that SCD knockdown restored ER and Golgi dilation. Therefore, we propose a model in which D-2HG production increases hSCD expression within the ER, which leads to increased levels of MUFA-phospholipids, and ER and Golgi dilation (Fig. 7). This MUFA upregulation and phospholipid imbalance was not present in IDH1^*WT*^ cells, which showed normal morphology for these organelles (Fig. 7). Finally, we showed that tilting the phospholipid imbalance towards more MUFA leads to cell death more readily in IDH1^*mut*^ compared with IDH1^*WT*^ cells. Collectively, these findings identify a unique metabolic rewiring that produces a targetable vulnerability specific to IDH1^*R132H*^ tumors.

These results link the ER and Golgi-specific lipidomic profile of IDH1^*mut*^ gliomas with their altered organelle morphology. Although, imbalances in SFAs, MUFA and PUFAs have been linked to several diseases, including cancer^38,39^, this imbalance was unknown in the context of IDH1^*mut*^ gliomas. One prominent feature of this phospholipid imbalance is altered membrane fluidity, i.e., membranes with higher PUFA display greater fluidity^40^. We hypothesize that the accumulation MUFAs leads to increased membrane fluidity, resulting in increased trafficking of proteins and lipids though those membranes and potentially the burden on ER and Golgi. Our hypothesis is confirmed by the proteomic analysis, which revealed membrane trafficking as a common upregulated pathway among cell lines and tissue with high significance. Although the link between altered lipidome and Golgi morphology via the membrane trafficking^41,42^ has been shown before, the role of this pathway in the cancer setting has not been explored.

Understanding the role of altered lipid metabolism in maintaining the hallmarks of cancers is a relatively new area of investigation. While stearyl-CoA-desaturases have been shown to play crucial roles in other cancer types by modulating different signaling pathways^43^, their role in both organelle integrity and brain tumors remains unknown. Herein, by identifying the cellular consequences of upregulating these enzymes, we are opening a new direction into the role of these enzymes in organelle integrity. Furthermore, our results revealing that oleic acid leads to IDH1^*mut*^ specific cell death, offer new avenues to be explored in the search of targeted therapies for glioma. Since many of existing cancer therapeutics are not able to cross the blood–brain barrier^44^, the ability of oleic acid or analogous fatty acids to penetrate this barrier and reach the site of tumor, makes our approach viable for clinical application to IDH1^mut^ tumors^45^.

## Methods

### Cell models and culture

TS603 (grade III oligodendroglioma), BT142 (grade III astrocytoma) and NCH1681 (grade III astrocytoma)^46^ were grown in DMEM/F12 medium (Gibco, Thermo Fisher Scientific, Waltham, MA) supplemented with 1% N2 growth supplement (Thermo Fisher Scientific, Waltham, MA), heparin sulfate (Sigma-Aldrich, St. Louis, MO), penicillin-streptomycin (Gibco, Thermo Fisher Scientific, Waltham, MA), EGF and FGF from R&D Systems (Minneapolis, MN). U251 cells were obtained from Sigma-Aldrich (St. Louis, MO). Cells were cultured in DMEM high glucose (Thermo Fisher Scientific, Waltham, MA). supplemented with 10% fetal bovine serum (Thermo Fisher Scientific, Waltham, MA). The lentivirus was packaged in HEK293T cells using pMD2.G (Addgene cat #12259) and psPAX2 (Addgene cat #12260) system. U251^*WT*^ and U251^*R132H/C*^ cells were obtained via lentivirus transduction of the doxycycline-inducible pLVX-TetOne-Puro vector (Clontech, Mountain View, CA) carrying IDH1 R132C or R132H variants into U251 MG cells. Stable expression cell lines were selected via addition of puromycin (1–2 μg/mL), side by side with control cells^26^. NHA-EV, NHA^*WT*^ and NHA^*R132H*^ were grown in DMEM high glucose medium (Thermo Fisher Scientific, Waltham, MA) supplemented with penicillin-streptomycin (Gibco, Thermo Fisher Scientific, Waltham, MA), 10% FBS (Thermo Fisher Scientific, Waltham, MA) and 100 μg/ml hygromycin B from (Thermo Fisher Scientific, Waltham, MA).

### Cells for organelle Raman experiments

Untreated and AGI5198 treated cells (U251^*WT*^, U251^*R132H*^, and U251^*R132C*^) were seeded in the luminescence free 35 mm glass bottom dishes (Fisher Scientific Co, Hanover Park IL) using media described above. Right before the experiments, media was replaced with the warmed optically transparent DMEM media (Life Technologies Corporation, Grand Island NY) and dishes were covered with a 22 mm circle cover glasses (VWR Scientific, Chicago, IL) and sealing with a waterproof silicon sealant. To determine organelle-specific Raman spectra, we labeled each type of organelle, one at a time, with the following markers, MitoTracker Green FM (Life Technologies Corporation, Grand Island NY) for mitochondria, ER-Tracker Green (Life Technologies Corporation, Grand Island NY), for Endoplasmic Reticulum, and NBD C6 ceramide-BSA (Life Technologies Corporation, Grand Island NY) for Golgi Apparatus, following the manufacturer’s instructions. At the end of labeling, the cells were washed with warmed, sterile PBS three times and then the optically transparent DMEM supplemented with 25 mM of HEPES was added. Optimal labeling time was confirmed via confocal microscopy^25^.

### Raman spectroscopy

We used the DXR2 Raman microscope from Thermo Fisher Scientific (Madison, WI) which was equipped with a 633 nm@70 mW single frequency laser diode (ROUSB-633-PLR-70-1, Ondax, CA), and was set to deliver ~30 mW power to the live organelles, in order to preserve the live of the cells and eliminate the background noise. The machine was also equipped with a fluorescence Illuminator (5-UR7005) with a green fluorescence cube (488/561EX) and a mercury lamp (X-Cite 120 PC, Photonic Solutions, Inc., MA). To acquire the data, the microscope was connected with OMNIC software for dispersive Raman (Thermo Fisher Scientific, Madison, WI) and to quantify different classes of biomolecules, the computer had the BCAbox software (ACIS, LLC, Buffalo, NY) installed. In this setup a plan N oil immersion 100^x^ (N_a_ = 1.25) Olympus objective lens was used because it provides the submicron XY spatial resolution and it produces a lower background signal compared to that of a water immersion with similar numerical aperture. Raman spectral concentration calibration was performed using bovine serum albumin, calf thymus DNA, S. cerevisiae RNA (Sigma–Aldrich, St. Louis, MO, USA), and bovine heart lipids (Avanti Polar Lipids, Alabaster, AL, USA), with unit weight of 100 mg/mL for proteins and 20 mg/mL for RNA, DNA and lipids concentrations. The BCA toolbox yields a set of biomolecular weights (e.g., proteins, DNA, RNA, lipids, and glycogen) and residual profile for each analyzed spectrum. Representative BCA processed spectra and output data are shown in Fig. 1. Cells were treated for 48 h and subsequently media was removed and media without phenol red was added for Raman measurements which lasted about 1 h^25^. Each data point is an average of at least three measurements. At least 15 data points were taken for each organelle.

### Organelle collection

Adherent cells were grown in triplicate to a confluence ≥90% in a large-sized T175 tissue culture flask. About 20 flasks were used per variant (U251^*WT*^, U251^*R132H*^, U251^*R132H*^ + 12.5 μM AGI5198). To each flask was administered 3.0 mL 0.05% Trypsin and incubated for 1 min. Pipetting was conducted to dissociate adherent cells from plate surfaces. The homogenous cell media was transferred to sterile conical tubes and centrifuged at 400 × *g* for 5 min at room temperature. Supernatant was discarded and pellet was washed with 1.0 mL PBS. Cells were centrifuged 40 × *g* for 3 min at room temperature. Supernatant was completely aspirated and discarded. For tissue and/or cell pellets, approximatively 200 mg of wet cell pellet and/or tissue was used for the extraction of endoplasmic reticulum using ER/Golgi Extraction Kits from Invent Biotechnologies according to the manufacture instructions. At the end of organelle extraction, pellets containing ER or Golgi were quenched via snap freezing in dry ice and storing at −80 °C until metabolomic/lipidomic extraction and BCA protein quantification.

### Enzyme prediction

The differential enrichment analysis was performed via Metaboanalyst 4.0^47^ platform for metabolites presenting fold-change ≥1.4. The hypergeometric *p* values (p) were calculated based on probability of randomly selecting enzyme/pathway for over-representation analysis (ORA) given the predicted metabolite set and the total number metabolites/substrates associated with the enzyme/pathway. Fold enrichment (FE) was determined based on hits from the predicted metabolite set divided by the hits expected by chance. The −log (p) and (FE) were performed to scale and plot data in Excel; where p is represented by color and FE is represented by size.

### LC/MS biphasic extraction for polar metabolites and nonpolar lipids

Prior to sonication, tissue and cells samples were administered 500 µL ice-chilled MilliQ water and lysed via sonication by Misonix XL-2000 Ultra-liquid processor (Misonix Inc., Farmingdale, NY, USA) at 40 amps for 30 s. Ice-chilled MilliQ water was administered each organelle pellet derived from cells and tissue while maintaining samples in ice bath in order to quench throughout extraction procedure. Organelle pellets were lysed via sonication by Misonix XL-2000 Ultra-liquid processor (Misonix Inc., Farmingdale, NY, USA) at 40 amps for 30 s. Following sonication, a 40 μL aliquot of each cell lysate was collected and stored at −80 °C for Bradford protein assay to later normalize target analyte concentration to nmol/mg protein. Prior to extraction, 100 μL chilled 50% MeOH (aq) reagent spiked with qualitative internal standards (qIS) at 0.125 μM nitrodracylic acid (NDA) and 0.125 μM isocaramidine sulfate (ICS) was added to each lysate. Samples were vortexed on BenchMixer (Benchmark Scientific, Edison, NJ, USA) at mid-speed (6) for 15 s and incubated on ice for 10 min. Bligh and Dyer biphasic liquid extraction was performed upon the addition of 500 μL methanol chilled (−20 °C) LC/MS grade Methanol (MeOH) was added to each followed by 275 μL chilled (−20 °C) chloroform. Next, 100 µL chilled chloroform reagent spiked with qIS, phenyl-N-pyridinyl acrylamide (PNPA) was added to each lysate solution. Each solution was vortexed on BenchMixer (Benchmark Scientific, Edison, NJ, USA) at mid-speed (6) for 15 s and placed on rotating mixer at mid-speed for 60 min on ice bath to precipitate protein. The samples were centrifuged at 12,000 × *g* for 18 min at 4 °C. The resulting two phases (upper hydrophilic and lower hydrophobic lipid) were separated and collected as well as the protein disk. Metabolite extracts were concentrated under N_2_ gas flow on Techne sample concentrator with PTFE-coated needles (Cole-Palmer, Vernon Hills, IL, USA) at 25 °C until completely dry, snap frozen and then stored at −80 °C, while the protein pellet resulting from extraction was stored in −80 °C for further proteomics analysis.

### Independent nonpolar and polar lipidomic profiling using ultra high-performance liquid chromatography and quadrupole time-of-flight mass spectrometry (UHPLC-QTOF-MS) LC/MS

In order to detect and quantify the nonpolar lipidome (fatty acids, acylcarnitines, fatty acid primary amide, eicosanoids, prostaglandins, amino-acyl endocannabinoids, triglycerides, ceramides, glycoceramides, sulfatide as well as numerous classes of glycerophospholipids—PE, LPE, PC, LPC,,PS, LPS, PG, PIP), the hydrophobic lower phase of organelle extracts from cell and tissue were reconstituted in 80 µL reagent containing methanol/ACN/water ratio 50:25:25^48^. Pooled quality control (QC) samples were composed of 10% volume from each sample. LC/MS lipidomic analysis was performed on acquired on the Agilent 6545 quadrupole time-of-flight mass spectrometer coupled with Infinity II 1290 liquid chromatography ultra high-pressure system. Lipids in the nonpolar extracts were separated using Acquity UPLC CSH 1.7 µm, 2.1 × 100 mm C18 column (Waters Corp. Mass., USA, 186005297) composed of mobile phase gradient consisted of ACN/water (30/70) containing 5 mM ammonium formate and 0.1% formic acid (Solvent A) and IPA/ACN (90/10) containing 10 mM ammonium formate and 0.1% formic acid (Solvent B) while maintaining isothermal column temperature of 45 °C and static flow rate of 0.250 mL/min. Real-time mass correction was applied with 0.2 mL/min infusion of external standard (containing TFA/PURINE/HP921) in 95:5 ACN/water. Data were acquired across mass-to-charge (m/z) range, 75–1300 *m/z*. For electrospray injection (ESI) negative ion, sample extracts were injected (7.5 µL) and resolved using gradient timetable: 0–0.1 min, 1% B; 0.8 min, 5% B, 2.0 min, 35% B; 4.5 min 38% B; 4.75 min, 40% B; 7.5 min, 71% B; 8.5 min, 80.5% B; 9.5 min, 91.5% B; 11 min, 98% B; 12.25 min, 100%; 12.5 min, 99 % B; 13.5 min, 1 %B equilibrate, 1.5 min. The following MS parameters were applied: drying gas temperature (temp), 150 °C; gas flow 7 L/min; nebulizer, 45 psi; sheath gas temp, 350 °C; sheath gas flow, 12 L/min; capillary voltage, 3000 V; nozzle voltage, 25 V; fragmentor, 90 V; skimmer, 50 V; scan rate, 3.0 spectra/sec. Alternatively, ESI-positive acquisition injected (8.0 µL) and resolved using gradient timetable: 0–0.1 min, 1% B; 0.3 min, 5% B, 1.75 min, 35% B; 4.25 min 38% B; 4.5 min, 40% B; 7.25 min, 71% B; 9.25 min, 98% B; 10.25 min, 100% B; 10.5 min, 99% B; 11.25 min, 1% B equilibrate, 1.5 min. The following MS parameters were applied: injection volume, 6.5 µL; drying gas temp, 150 °C; gas flow 6 L/min; nebulizer, 40 psig; sheath gas temp, 350 °C; sheath gas flow, 12 L/min; capillary voltage, 3500 V; nozzle voltage, 35 V; fragmentor, 150 V; skimmer, 50 V; scan rate, 3.5 spectra/s.

The upper phase containing polar extracts was resuspended in 60% methanol (aq). LC/MS data acquisition was conducted with zwitterionic HILIC (HILIC-Z) assays to achieve broad detection and high resolution of central carbon metabolites and polar lipids. Amino acids, nucleotides, carbohydrates, pyrimidine, ribonucleotides, free fatty acids, branched-chain fatty acids, diglycerides, sphingomyelin as well as glycerophospholipids—PA, PI, PG—were among the polar metabolite and lipid classes that were separated using the InfinityLab Poroshell 120 HILIC-Z 2.1 ×100 mm, 2.7 µm column (Agilent Technologies, Ca., USA, 685775-924). HILIC-Z column acquisition was performed ESI negative mode. Compounds were resolved over a gradient composed of mobile phase A—10 mM ammonium acetate in 88% water and 12% acetonitrile, pH 6.85—and mobile phase B—10 mM ammonium acetate in 90% acetonitrile (aq)—using a constant column temperature 30 °C. The gradient was applied at flow rate 0.24 mL/min: 100% B, 0.5 min; 95% B, 2.0 min; 60% B, 3.0 min; 35% B, 5 min; hold 0.25 min; 0% B, 6 min; hold 0.5 min; 100% B, 7.25 min. Real-time mass correction was applied with 0.2 mL/min infusion of external standard (containing TFA/PURINE/HP921) in 95:5 ACN/water. The mass analyzer parameters included drying gas temperature 250 °C, sheath gas temperature 325 °C, nebulizer 45 psi, skimmer 50 V, octopole radio frequency 750 V and scan rate of 4 spectra/s. In ESI-positive mode experiment, MS spectra were acquired over a voltage gradient of capillary 3500 V, nozzle 2000 V, and fragmentor 165 V. In ESI negative mode experiment, mass spectra were acquired over a voltage gradient of capillary 3000 V, nozzle 1500 V, and fragmentor 80 V. Throughout all assay conditions, only LC/MS grade solvents and additives were used to prepare reagents, mobile phases and wash solutions, unless otherwise indicated. Wash cycles consisting of strong wash (50% Methanol, 25% Isopropanol, and 25% Water), and seal wash (10% Isopropanol and 90% water) were utilized to eliminate carryover for consecutive injections. Extract injections were randomized for samples to avoid analytical bias.

### LC/MS lipidomic data analysis

Prior to preprocessing each dataset, pooled QC samples (TIC, BPI, and EIC) were chromatographically examined to inspect consistency of retention time and ionization levels throughout. Following acquisition, mass feature bins were defined by partitioning the *m/z* vs. retention time (RT) matrices into fixed width using Agilent Masshunter Profinder B.08.00. Bins were manually inspected to confirm consistent, reproducible integration for each compound of interest across all samples. Precursor m/z for each bin was determine using molecular feature extraction algorithm to deconvolute, integrate, and envelope parent ions, adducts (H−, Cl+, H+, Na+), natural isotopes and neutral losses to define each composite spectrum. Logical binning and mass identification were conducted via targeted ion selection, annotation and alignment restricted to accurate neutral mass ±5.0 mDa with retention time ±0.4 min of references defined in Personal Compound Data Library (PCDL) for polar and nonpolar features including lipids. The PCDL contained putative identities and retention times for lipid that were defined using untargeted molecular feature extraction for pooled QC sample. Follow-on database search with LipidMaps and BioCyc, Kegg, HMDB were conducted to assign identities to the exact mass (±5.0 mDa) determined for each feature. This approach allowed for the targeting of select features in our analysis. Following preprocessing, raw abundance values were normalized to sample-specific internal standard response and corrected sample-specific protein quantification (as determined via BSA protein analysis). The qualitative internal standard NDA and ICS were utilized to normalize the ESI negative and positive mode data, respectively, for polar extractions; while PNPA was utilized to normalize abundance for both ESI ±data (primarily, lipids) acquired from nonpolar extracts. Normalized abundance data acquired for fatty acid and other lipids detected in both polar and nonpolar phases were combined for further comparative analysis. General features abundances have been reported in data repository while in depth analysis was reserved for specific features of interests (including MUFA, PUFA and PCs) and reported herein based on Raman spectroscopy directed discovery. Compound features containing zero values were eliminated to reduce bias. Graphs were generated in Prism 8.0. For each graph, the data are represented as the mean normalized abundance (counts per sec/µg protein in sample) using error bars as a report of standard deviation. Raw *p* values were represented as stars (*p* ≤ 0.05, *p ≤ 0.005,**p ≤ 0.005, ***; not significant ≈ no star, or ns). Statistical analysis utilized MetaboAnalyst 4.0^47^ online tools for the student t-test assuming equal variance.

### Protein digestion and TMT labeling

The fractionated subcellular fractionated pellets were solubilized in 50 mM HEPES, pH 8.0 containing 20% methanol. Digestion was performed by addition of trypsin at a ratio of 1:50 (Promega) and incubating overnight at 37 °C. Digestion was acidified by adding formic acid (FA) to a final concentration of 1%. The digest was desalted using Pierce peptide desalting columns according to manufacturer’s protocol. Peptides were eluted from the columns using 50% ACN/0.1% FA, dried in a speedvac and kept frozen in −20 ^o^C for further analysis. The concentration of the peptide was estimated using Pierce Quantitative Fluorescent peptide assay kit. For TMT labeling 25 ug of each sample was reconstituted in 50 ul of 50 mM HEPES, pH 8.0, and 100 ug of TMT label in 100% ACN was added to each sample. After incubating the mixture for 1 hr at room temperature with occasional mixing, the reaction was terminated by adding 8 ul of 5% hydroxylamine. The peptide samples for each subcellular fraction were pooled and speedvac to dry labeled peptide sample. The samples were cleaned up using peptide desalting columns.

### High pH reverse phase fractionation

The first dimensional separation of the peptides was performed using a Waters Acquity UPLC system coupled with a fluorescence detector (Waters, Milford, MA) using a 150 × 3.0 mm Xbridge Peptide BEM^TM^ 2. 5 um C18 column (Waters, MA) operating at 0.35 ml/min. The dried peptides were reconstituted in 100 ul of mobile phase A solvent (3 mM ammonium bicarbonate, pH 8.0). Mobile phase B was 100% acetonitrile (Thermo Fisher). The column was washed with mobile phase A for 10 min followed by gradient elution 0–50% B (10–60 min) and 50–75%B (60–70 min). The fractions were collected every minute. The fractions collected along the fractionation were pooled into 24 fractions, vacuum centrifuged to dry and stored at −80 ^o^C until analysis by mass spectrometry.

### Mass spectrometry proteomics acquisition and data analysis

The dried peptide fractions were reconstituted in 0.1% TFA and subjected to nanoflow liquid chromatography (Thermo Easy nLC 1000, Thermo Scientific) coupled to high resolution tandem MS (Q Exactive, HF, Thermo Scientific). Peptides were separated using a second dimension low pH gradient using a 2–40% ACN over 120 min in mobile phase containing 0.1% formic acid at 300 nl/min flow rate. MS scans were performed in the Orbitrap analyser at a resolution of 120,000 with an ion accumulation target set at 3e^6^ and max IT set at 50 ms over a mass range of 200–1800 *m/z*, followed by MS/MS analysis at a resolution of 45,000 with an ion accumulation target set at 1e^5^, max IT of 120 ms and first fixed mass set at 105 *m/z*. MS2 precursor isolation width was setup at 0.7 *m/z*, normalized collison energy was 29, and charge state 1 and unassigned charge states were excluded. Acquired MS/MS spectra were searched against a human uniprot protein database along with a contaminant protein database, using a SEQUEST and percolator validator algorithms in the Proteome Discoverer 2.2 software (Thermo Scientific, CA). The precursor ion tolerance was set at 10 ppm and the fragment ions tolerance was set at 0.02 Da along with methionine oxidation included as dynamic modification and TMT6 plex (229.163 Da) set as a static modification of lysine and the N-termini of the peptide. Trypsin was specified as the proteolytic enzyme, with up to two missed cleavage sites allowed. Searches used a reverse sequence decoy strategy to control for the false peptide discovery and identifications were validated using percolator software.

### Quantification of ER and Golgi

Images containing either ER and Golgi were imported into Image J. The scale was set for each image and converted into nm. Using the measuring tool in Image J, the distance from two adjacent membrane was measured and stored as a CSV file. The distances were imported into Prism 8.2.1 where statistical analysis and plotting was conducted.

### Proteomic data analysis

Ratio of protein abundances obtained from the proteomic analysis were used to create fold changes of the three specific groups: Patient 1^*Tumor/Margin*^, U251^*R132H/WT*^, and U251^*R132C/WT*^. More than 5500 protein IDs together with the fold changes of their expression were submitted to Reactome webserver and the report data was used to create graphs of pathways versus p value for each comparison.

### Confocal microscopy

A Zeiss LSM880 confocal microscope equipped with a ×63 plan-apochromat (N.A. 1.4) oil immersion objective lens, a PeCon stage top incubator to control temperature (37C), humidity and CO2, and a Bioptechs objective lens heater was used to acquire confocal images of live U251 IDH1 mutant and IDH^WT^ cells labeled with RFP expressing proteins in different organelles and C16 BODIPY for saturated fatty acid colocalization. Confocal images were collected with 2× frame averaging, 1.0 um optical section thickness and 0.09 um X–Y pixel size. A corresponding differential interference contrast (DIC) image was also acquired. A Nikon Ti2-E microscope equipped with a Yokogawa CSU-W1 spinning disk confocal unit, a ×60 plan-apochromat (N.A. 1.4) oil immersion objective lens and Hamamatsu ORCA Flash 4.0 V3 sCMOS camera was used to acquire images of live U251^*WT*^, U251^*R132H*^, U251^*R132C*^ cells labeled with lysotracker and in absence and presence of IDH1^*mut*^ inhibitor, AGI5198. Time-lapse confocal images were collected every second over a 120 s time period with 0.1 um X–Y pixel size. A Zeiss Elyra 7 lattice structured illumination microscope equipped with a ×63 alpha plan-apochromat (N.A. 1.46) oil immersion objective lens and dual PCO Edge 4.2 sCMOS cameras was used to acquire z-stacks of U251^*R132H*^ cells labeled with Golgi RFP and C16-BODIPY. Images were collected using fast frame switching in lattice SIM mode with 0.1 um z-step size, 0.03 um X–Y pixel size, and processed using the SIM module of the Zen software.

### Tissue sample preparation for TEM

Fresh tissue was stored immediately in formaldehyde (4% v/v), glutaraldehyde (2% v/v) cacodylate buffer (0.1 M, pH 7.4) until ready to be analyzed. Tissue specimens were transferred to wax paper and cut into 3 mm small pieces, which were stored in the fixative for 2 h at room temperature. Processing and embedding were carried out at room temperature in a fume hood. The cells were washed two times in cacodylate buffer for 10 min each prior to post-fixation of 1 h in osmium tetroxide (1% v/v) in the dark. After which the tissue was rinsed with twice with distilled water followed by cold sodium acetate buffer (0.1N, pH 4.5). en bloc stain in 0.5% w/v uranyl acetate (0.5% v/v) in acetate buffer (0.1N, pH 4.5) for 1 h and in the dark to prevent the uranyl acetate from being precipitated. The tissue is then washed with changes of acetate buffer for 10 min each and dehydrated sequentially with multiple washes of ethanol solution (35, 50, 75, 95, and 100%). Following three washes of 100% ethanol the tissue was rinsed again with 100% propylene oxide for 10 min each at room temperature followed by mixing with a 50/50 mixture (1:1 epoxy resin and propylene oxide mixture) overnight on a rotor in a chemical fume hood at room temperature and cured overnight. The following day the tissue was washed three times with pure epoxy resin prior to embedding. Once embedded the resin was cured for 48 h at 55 °C. Cured blocks were trimmed and thin sectioned at 70–90 nm using an ultramicrotome (Leica (Bannockburn, IL) and transferred onto copper mesh grids. Grids were stained with 0.5% uranyl acetate for 90 s followed by washing with distilled water and lead citrate for 90 s followed by washing with distilled water. Stabilization of grids was done by carbon evaporation in a vacuum evaporator followed by imaging on TEM. The grids were scanned and imaged at high and low magnification in the electron microscope operated at 80 kv. A CCD camera captured the digital images^49,50^.

### Cell samples preparation for TEM

The IDH1^*R132C*^ IDH1^*R132H*^, IDH1^*WT*^ cells and their derivatives were cultured in six-well plates and fixed in formaldehyde (4% v/v), glutaraldehyde (2% v/v) cacodylate buffer (0.1 M, pH 7.4) for at least 2 h in preparation for thin-sectioned EM analysis^49,50^. Processing and embedding were carried out at room temperature in a fume hood. The cells were washed two times in cacodylate buffer prior to post-fixation of 1 h in osmium tetroxide (1% v/v). *en bloc* stain in 0.5% w/v uranyl acetate (0.5% v/v) in acetate buffer (0.1 M, pH 4.5) for 1 h and in the dark to prevent the uranyl acetate from being precipitated. The cells were dehydrated through a series of multiple washes of ethanol solution (35, 50, 75, 95, and 100%). Following three washes of 100% ethanol, the cells were washed with pure epoxy resin overnight and washed two more times the following day prior to embedding. Once embedded the resin was cured for 48 h at 55 °C. The cured resin blocks were separated from the plate by submerging in liquid nitrogen. In preparation for thin sectioning, the separated resin blocks were examined under an inverted microscope to select an area with a large number of cells. The selected area was thin sectioned at 70–90 nm using an ultramicrotome equipped with a diamond knife and transferred onto copper mesh grids. Similar to tissue, grids were stained with 0.5% uranyl acetate and lead citrate, stabilized by carbon evaporation and imaged by TEM. The grids were scanned and imaged at high and low magnification in the electron microscope operated at 80 kv. A CCD camera captured the digital images^49,50^. Hitachi 7600 and Hitachi 7650 TEM was used for data acquisition equipped with a 2k × 2k AMT software.

### Time course of viability after treatment with oleic acid

For measurement of time course of viability after treatment with oleic acid for patient-derived glioma cell lines, cells were seeded as single cell suspension at a concentration of 100,000 cells/well in 48-well plates. Cells were treated with oleic acid (Sigma Cat#75090) at concentrations of 100 µM or DMSO as control. Cell viability was measured using Vi-CELL XR (Beckman Coulter) according to the manufacturer’s protocol at 24, 48, and 72 h after addition of the oleic acid. All assays were performed in five replicates.

### Sensitivity of patient-derived glioma cell lines to oleic acid (using Vi-CELL XR)

Various types of patient-derived glioma cell line were seeded as single cell suspension at a concentration of 100,000 cells/well in 48-well plates. Cells were treated with oleic acid (Sigma Cat#75090) at concentrations ranging from 100 µM to 500 µM or DMSO as control and incubated for 72 h. Cell viability was measured using Vi-CELL XR (Beckman Coulter) according to the manufacturer’s protocol. All assays were performed in five replicates.

### EC_50_ determinations

Sensitivity of patient-derived glioma cell lines to oleic acid was detected using the cell viability, CCK-8 kit. Various types of patient-derived glioma cell line were seeded as single cell suspension at a concentration of 20,000 cells/well in 96-well plates. Cells were treated with oleic acid (Sigma Cat#75090) at concentrations ranging from 20 µM to 400 µM or DMSO as control and incubated for 72 h. Relative cell numbers were quantified by the CCK-8 assay (Dojindo Molecular Technologies, Rockville, MD) according to the manufacturer’s protocol. Assays for BT142 and TS603 were performed in five replicates while for GSC827 and NCH1681 in four replicates.

### Apoptosis assay using flow cytometry

Apoptosis was assessed using PE Annexin V Apoptosis Detection Kit I (BD Biosciences) and analyzed by flow cytometry. Briefly, cells were plated into six-well culture dishes (1 × 10^6^ cells/well) for 24 h. prior to the addition of oleic acid (Sigma Cat#75090) at a concentration of 150 µM. Following 24 hrs incubation with oleic acid the percentage of apoptotic cells was determined by the annexin V-PE/7-AAD assay following manufacturer’s instructions. Fluorescence of the cells was immediately determined by a Sony SA3800 spectral analyzer.

### SiRNA

5 × 10^5^ cells/well in six-well plates were transfected with a set of three siSCD1 specific-targeting small interfering RNAs (OriGENE Inc, SR304248) using siTran 2.0 siRNA transfection reagent (OriGENE Inc, TT320001) for 18 h following the manufacturer’s protocol. Trilencer-27 Fluorescent-labeled transfection control siRNA duplex (OriGENE Inc, SR30002) were used as controls. After three days incubation, cells were treated again with specific-targeting siRNA or control siRNA for 2 days and then analyzed.

### RT-PCR

Total RNA was extracted by using RNeasy spin columns (Qiagen, Chatsworth, CA, USA; cat. no.: 74136). After treatment with RNase-free DNase (Qiagen), RT was performed on RNA by using Clontech RNA to cDNA EcoDry kit (cat.no.: 639548) according to the manufacturer’s protocol. RT-qPCR was performed on QuantStudio. Reactions were carried out in 10 *μ*L by using SYBR Green PCR master mix according to the manufacturer’s protocol (Bio-Rad, cat. no.: 1725017, Hercules, CA). The concentration of primer pairs was 100 nM. All primer sequences are shown in the primer table. All reactions were performed in triplicate. To make comparisons between samples and controls, the CT (cycle threshold, defined as the cycle number at which the fluorescence is above the fixed threshold) values were normalized to the CT of *beta*-actin in each sample. The following primers were used hSCD1-F: 5’-AAACCTGGCTTGCTGATG-3’; hSCD1-R: 5’-GGGGGCTAATGTTCTTGTCA-3’; beta-actin-F: 5’-ACTGGAACGGTGAAGGTGAC-3’, beta-actin-R: 5’-GTGGACTTGGGAGAGGACTG-3’.

### Western blot analysis

The cellular proteins were purified from treated or control cells. Equal amounts of protein (10 μg) were loaded in each lane for NuPAGE 4–12% Bis‐tris gel and then transferred to polyvinylidene difluoride membranes. The membranes were washed with blotting buffer (1 × PBS containing 0.1% Tween20) and then blocked for 60 min in blotting buffer containing 10% low‐fat powdered milk. Membranes were washed three times with blotting buffer, incubated at 4 °C overnight with primary antibody (1:1000) containing 5% lowfat powdered milk, and incubated with HRP conjugated secondary antibody (1:1000) at room temperature for 60 min. The blots were detected with Bio-Rad image system. The relative expression of proteins was normalized to either β-actin (cat.no: 8457) purchased from Cell signaling Technology, of α-tubulin purchased from Abcam (ab15568). and analyzed using Image J. SCD-1 (cat.no: ab19862), FASN (cat.no: ab218306), and SCD-5 (cat no: ab130958) were purchased from Abcam.

### Patient samples

Tissue samples were obtained from the NOB clinic, from patients undergoing surgery at NIH and following the approval of the National Cancer Institute Institutional Review Board. All patients were required to sign an informed consent for these samples.

### Reporting summary

Further information on research design is available in the Nature Research Reporting Summary linked to this article.

## Supplementary information

Supplementary Information

Reporting Summary

## Data Availability

Data supporting the findings of this work are available within the paper and its Supplementary Information files. A reporting summary for this article is available as a Supplementary Information file. The metabolic datasets generated and analyzed in this study have been deposited in Metabolights database under the accession numbers, MTBLS1974, MTBLS1973; MTBLS1967. The proteomics dataset generated in this study has been deposited in to MassIVE under the accession number, MSV000085841. Reactome database was used for proteomics analysis. Source data are provided with this paper. The remaining data are available within the Article, Supplementary Information files or are available from the authors upon request.

## Source data

Source Data
