# Supplementary material for: IDH1 mutations induce organelle defects via dysregulated phospholipids
Source: Nat Commun. 2021 Jan 27;12:614. doi: 10.1038/s41467-020-20752-6 (PMC7840755; doi:10.1038/s41467-020-20752-6)
Supplement: Supplementary file 1 — Supplementary Information [file 41467_2020_20752_MOESM1_ESM.pdf]

Supplementary for **"IDH1 Mutations Induce Organelle Defects Via Dysregulated Phospholipids"**

Contact for Reagents and Resource Sharing. For any material used in the study, please contact Mioara Larion at: mioara.larion@nih.gov

Supplementary Table 1. List of antibodies and reagents used

REAGENT or RESOURCE	SOURCE	IDENTIFIER
**Antibodies**		
SCD-1	Abcam	ab19862
SCD-5	Abcam	ab130958
FASN	Abcam	ab218306
α–tubulin	Abcam	ab15568
β-actin (D6A8) Rabbit	Cell signaling Technology	8457
**Chemicals, Peptides, and Recombinant Proteins**		
Octyl-R-2HG	Sigma-Aldrich	SML2200-25MG
Fatostatin A	Tocris	4444
Bodipy-C16	ThermoFisher	D3821
Halt™ Protease and Phosphatase Inhibitor Cocktail (100X)	ThermoFisher	78442
Palmitoleic Acid	Cayman Chemicals	10009871
Oleic Acid	Sigma-Aldrich	75090-5ML
CAY10566	Cayman Chemicals	944808-88-2
Linoleic acid	Sigma-Aldrich	L1012-1G
Cerulenin	Sigma-Aldrich	C2389-5MG
CellLight™ Golgi-RFP, BacMam 2.0	ThermoFisher	C10504
CellLight™ Lysosomes-RFP, BacMam 2.0	ThermoFisher	C10504
CellLight™ Mitochondria-RFP, BacMam 2.0	ThermoFisher	C10601
CellLight™ ER-RFP, BacMam 2.0	ThermoFisher	C10591
ER-Tracker™ Green(BODIPY™ FL Glibenclamide)	ThermoFisher	E34251
Oil Red O solution	Sigma-Aldrich	O1391-250ML
HCS LipidTOX™ Green Neutral Lipid Stain	ThermoFisher	H34475

LysoTracker Red DND-99	ThermoFisher	L7528
Trilencer-27 Fluorescent-labeled transfection control siRNA duplex - 1 nmol	ORIGENE	SR30002
SCD1 (SCD) Human siRNA Oligo Duplex	ORIGENE	SR304248
SCD1 Human siRNA	Sigma-Aldrich	EHU108071-20ug
**Critical Commercial Assays**		
Pierce™ BCA Protein Assay Kit	ThermoFisher	23225
MINUTE™ GOLGI APPARATUS ENRICHMENT KIT	Invent Biotechnologies	GO-037
Minute™ ER Enrichment Kit	Invent Biotechnologies	ED-028
Minute™ Lysosome Isolation Kit	Invent Biotechnologies	LY-034
Cell Counting Kit-8	Dojindo Molecular Tech.	CK04-13
Annexin V PE Apoptosis Detection Kit	BD Biosciences	559763
FITC Annexin V Apoptosis Detection Kit with 7-AAD	BD Biosciences	640922
**Experimental Models: Cell Lines**		
TS603	MSKCC	N/A
BT142	ATCC	ATCC® ACS-1018™
GSC827	NOB	N/A
GSC923	NOB	N/A
U251	Sigma Aldrich	N/A
NCH1681	University of Heidelberg	N/A
**Software and Algorithms**		
Prism	Graphpad	https://www.graphpad.com/scientific-software/prism/
R	R Project for Statistical Computing	https://www.r-project.org/
Metaboanalyst	McGill University	http://www.metaboanalyst.ca
MassHunter Quant	Agilent	
Agilent Masshunter Profinder	Agilent	

Partek Genomic Suite	Partek	http://www.partek.com/introducing-partek-genomics-suite-version-70
Thermo Scientific™ OMNIC™xi Software	ThermoFisher	https://www.thermofisher.com/order/catalog/product/IQLAADGABFFAHCMBDI
BioRender	BioRender	https://biorender.com
BCAbox Software	ACIS, LLC	http://acis-us.com/

Supplementary Table 2. Fifhteen most significant pathways derived from proteomic analysis of Golgi extracts of tumor versus margin from Patient 1. A binomial test was used to calculate the probability shown for each result, and the p-values were corrected for the multiple testing (Benjamini–Hochberg procedure) that arises from evaluating the submitted list of identifiers against every pathway.

PATHWAY NAME	ENTITIES			
	Found	Ratio	p value	FDR
1. MEMBRANE TRAFFICKING	438/665	0.046	1.11e-16	7.89e-14
2. TRANSLATION	236/339	0.023	1.11e-16	7.89e-14
3. AXON GUIDANCE	357/584	0.04	1.11e-16	7.89e-14
4. METABOLISM OF RNA	447/782	0.054	7.77e-16	4.14e-13
5. SIGNALING OF ROBO RECEPTORS	175/235	0.016	1.89e-15	8.04e-13
6. NERVOUS SYSTEM DEVELOPMENT	367/620	0.043	3.77e-15	1.34e-12
7. REGULATION OF EXPRESSION OF SLITS AND ROBOS	141/183	0.013	8.97e-14	2.39e-11
8. EUKARYOTIC TRANSLATION INITIATION	107/130	0.009	1.96e-12	4.18e-10
9. CAP-DEPENDENT TRANSLATION INITIATION	107/130	0.009	1.96e-12	4.18e-10
10. GTP HYDROLYSIS AND JOINING OF THE 60S RIBOSOMAL SUBUNIT	101/120	0.008	2.33e-12	4.52e-10

11. LI3A-MEDIATED TRANSLATIONAL SILENCING OF CERULOPLASMID EXPRESSION	100/120	0.008	5.15e-12	9.12e-10
12. SRP-DEPENDENT COTRANSLATIONAL PROTEIN TARGETING TO MEMBRANE	99/119	0.008	7.19e-12	1.18e-09
13. MRNA SPLICING-MAJOR PATHWAY	135/185	0.013	1.15e-11	1.67e-09
14. ER-PHAGOSOME PATHWAY	124/165	0.011	1.29e-11	1.67e-09
15. PROCESSING OF CAPPED INTRON-CONTAINING PRE-MRNA	172/256	0.018	1.3e-11	1.67e-09

Supplementary Table 3. Fifhteen most significant pathways derived from proteomic analysis of Golgi extracts of U251^{R132C} Versus U251^{WT} cells. A binomial test was used to calculate the probability shown for each result, and the p-values were corrected for the multiple testing (Benjamini–Hochberg procedure) that arises from evaluating the submitted list of identifiers against every pathway.

**PATHWAY NAME**	**ENTITIES**			
	**Found**	**Ratio**	**p value**	**FDR**
1. TRANSLATION	238/294	0.026	1.11e-16	7.61e-14
2. MEMBRANE TRAFFICKING	421/635	0.056	1.11e-16	7.61e-14
3. METABOLISM OF RNA	461/675	0.059	1.11e-16	7.61e-14
4. SIGNALING OF ROBO RECEPTORS	168/218	0.019	4.51e-13	2.00e-10
5. EUKARYOTIC TRANSLATION INITIATION	109/120	0.011	5.85e-13	2.00e-10
6. CAP-DEPENDENT TRANSLATION INITIATION	109/120	0.011	5.85e-13	2.00e-10
7. REGULATION OF EXPRESSION OF SLITS AND ROBOS	140/172	0.015	9.79e-13	2.87e-10
8. GTP HYDROLYSIS AND JOINING OF THE 60S RIBOSOMAL SUBUNIT	103/113	0.01	2.05e-12	5.27e-10
9. LI3A-MEDIATED TRANSLATIONAL SILENCING OF CERULOPLASMID EXPRESSION	102/113	0.01	2.75e-12	6.26e-10
10. CELLULAR RESPONSES TO STRESS	348/564	0.049	4.27e-12	8.76e-10

11. NONSENSE-MEDIATED DECAY (NMD)	103/117	0.01	1.43e-11	2.45e-09
12. NONSENSE-MEDIATED DECAY (NMD) ENHANCED BY THE EXON JUNCTION COMPLEX (EJC)	103/117	0.01	1.43e-11	2.45e-09
13. CELLULAR RESPONSES TO EXTERNAL STIMULI	350/578	0.051	3.27e-11	5.15e-09
14. INFLUENZA INFECTION	134/171	0.015	3.53e-11	5.15e-09
15. FORMATION OF A POOL OF FREE 40S SUBUNITS	92/102	0.009	5.08e-11	6.96e-09

Supplementary Table 4. Fifhteen most significant pathways derived from proteomic analysis of Golgi extracts of $U251^{R132H}$ Versus $U251^{WT}$ cells. A binomial test was used to calculate the probability shown for each result, and the p-values were corrected for the multiple testing (Benjamini–Hochberg procedure) that arises from evaluating the submitted list of identifiers against every pathway.

**PATHWAY NAME**	**ENTITIES**			
	**Found**	**Ratio**	**p value**	**FDR**
1. TRANSLATION	238/294	0.026	1.11e-16	7.61e-14
2. MEMBRANE TRAFFICKING	421/635	0.056	1.11e-16	7.61e-14
3. METABOLISM OF RNA	461/675	0.059	1.11e-16	7.61e-14
4. SIGNALING OF ROBO RECEPTORS	168/218	0.019	4.51e-13	2.00e-10
5. EUKARYOTIC TRANSLATION INITIATION	109/120	0.011	5.85e-13	2.00e-10
6. CAP-DEPENDENT TRANSLATION INITIATION	109/120	0.011	5.85e-13	2.00e-10
7. REGULATION OF EXPRESSION OF SLITS AND ROBOS	140/172	0.015	9.70e-13	2.87e-10
8. GTP HYDROLYSIS AND JOINING OF THE 60S RIBOSOMAL SUBUNIT	103/113	0.01	2.05e-12	5.27e-10
9. LI3A-MEDIATED TRANSLATIONAL SILENCING OF CERULOPLASMID EXPRESSION	102/113	0.01	2.75e-12	6.26e-10
10. CELLULAR RESPONSES TO STRESS	348/564	0.049	4.27e-12	8.76e-10

11. NONSENSE-MEDIATED DECAY (NMD)	103/117	0.01	1.43e-11	2.45e-09
12. NONSENSE-MEDIATED DECAY (NMD) ENHANCED BY THE EXON JUNCTION COMPLEX (EJC)	103/117	0.01	1.43e-11	2.45e-09
13. CELLULAR RESPONSES TO EXTERNAL STIMULI	350/578	0.051	3.27e-11	5.15e-09
14. INFLUENZA INFECTION	134/171	0.015	3.53e-11	5.15e-09
15. FORMATION OF A POOL OF FREE 40S SUBUNITS	92/102	0.009	5.08e-11	6.96e-09

SUPPLEMENTARY FIGURES

Supplementary Fig. 1:

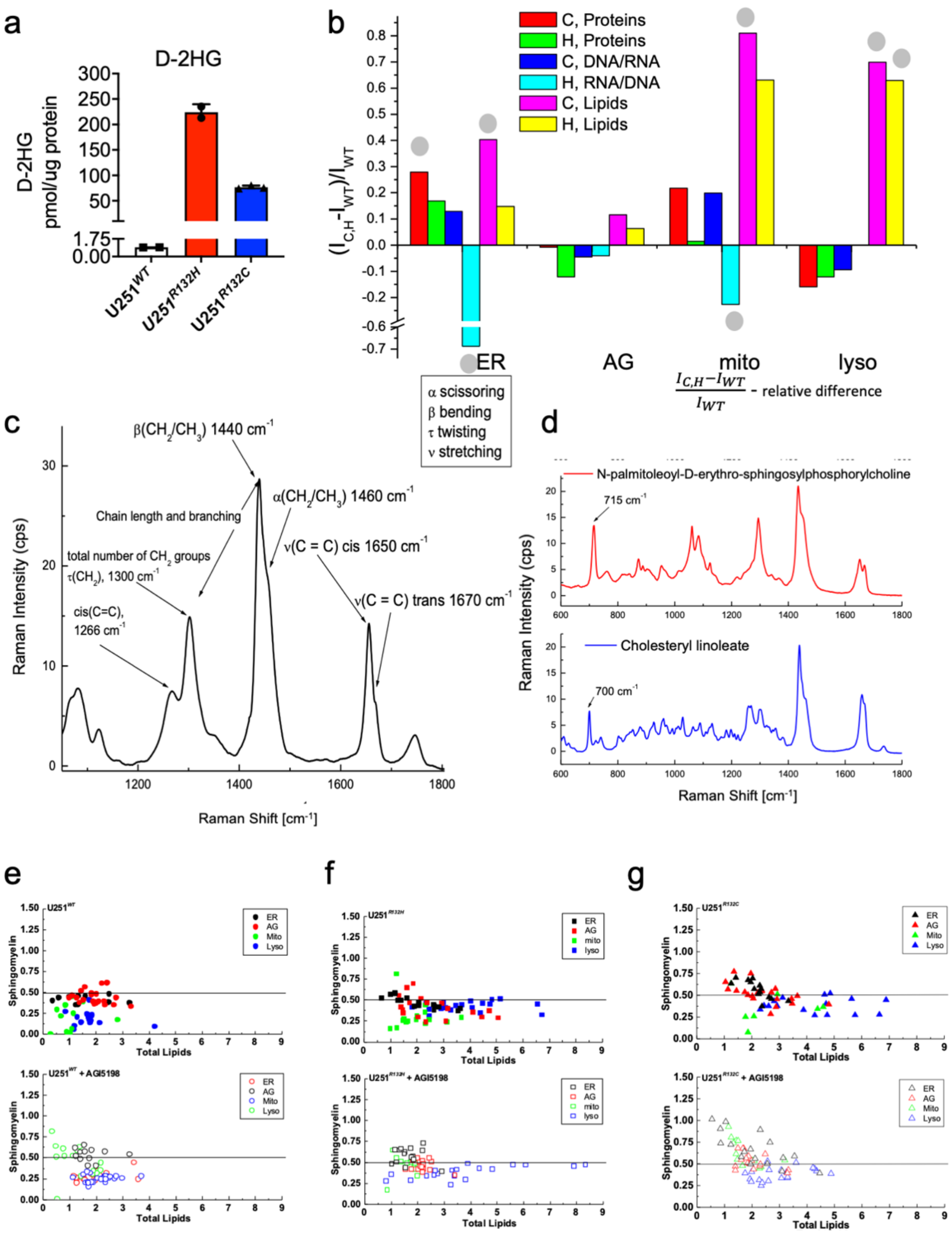

Supplementary Fig. 1: Detailed analyses of organelle specific Raman data shows that the composition of lipids is different in different mutants. a. Different mutants produce different concentration of D-2hydroxyglutarate as measured via LC/MS. Values were determined from biologically independent samples for U251^{R132C} (n=3), U251^{WT} (n=2) and U251^{R132H} (n=2) cells. Data are presented as

mean values +/-SD. **b.** Comparison of average levels of biomolecular components obtained from Raman analysis show differences due to the R132H, and R132C mutations. The levels above the x axis show increased levels in the U251$^{R132H/C}$ cells, while the bars below the x axis show increased levels in the U251^{WT} cells. C denotes U251^{R132C}, while H denotes U251^{R132H} cells. **c** and **d.** Raman spectrum depicting the assignment from which information regarding lipid structure is obtained such as the lipid unsaturation parameter and the Cis/Trans ratio and various Raman spectra for standards used. **e-g.** Sphingomyelin levels are heterogeneously distributed in organelles of both mutant cells (upper panels) and become more homogenous after addition of AGI-5198 inhibitor with the exception of lysosomes (lower panels). p values determined by unpaired two-tailed Student's t-test in **b**. p-values are represented as follows: ****, p<0.00005, ***, p < 0.0005; **, p<0.005; *, p<0.05; ns, not significant and were calculated using one-way ANOVA followed by Tukey's test for multiple comparisons. For simplicity, only significant values are shown on the graphs. Source data are provided as a Source Data file.

Supplementary Fig. 2:

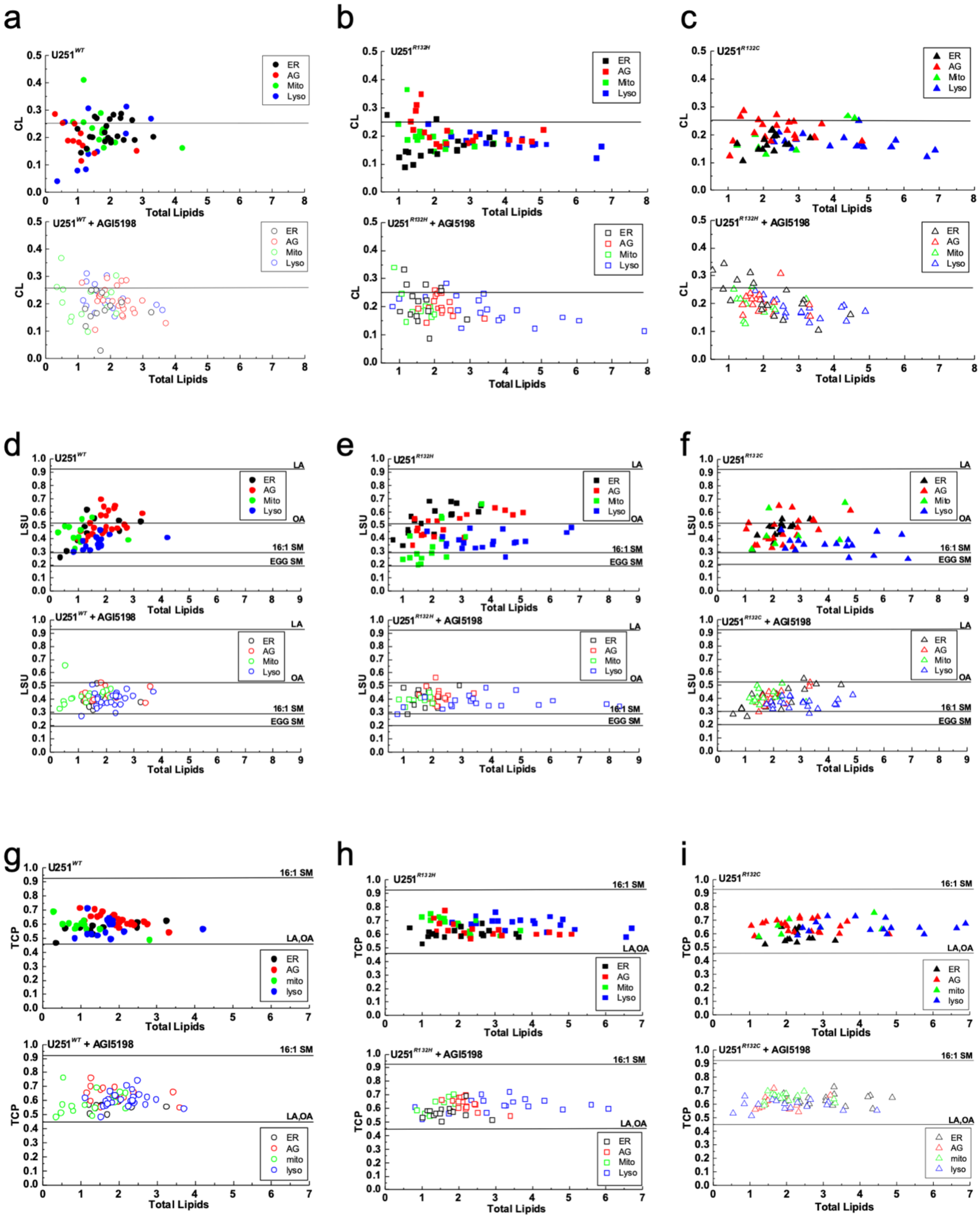

Supplementary Fig 2: Heterogenous distribution of lipid parameters across cells and organelles. a-c. Cholesterol levels are heterogeneously distributed in both U251^{R132H} and U251^{R132C} cells compared with the U251^{WT} cells (upper panels) and this distribution become more homogenous upon addition of AGI-5198 inhibitor (lower panels) with the exception of lysosomes. **d-f** LSU parameter is heterogeneously distributed in both U251^{R132H} and U251^{R132C} cells compared with the U251^{WT} cells (upper panels) and this distribution become more homogenous upon addition of AGI-5198 inhibitor (lower panels) with the exception of lysosomes. **g-i.** TCP parameter is heterogeneously distributed in both U251^{R132H} and U251^{R132C} cells compared with the U251^{WT} cells (upper panels) and this distribution become more homogenous upon addition of AGI-5198 inhibitor (lower panels) with the exception of lysosomes. Source data are provided as a Source Data file.

Supplementary Fig. 3:

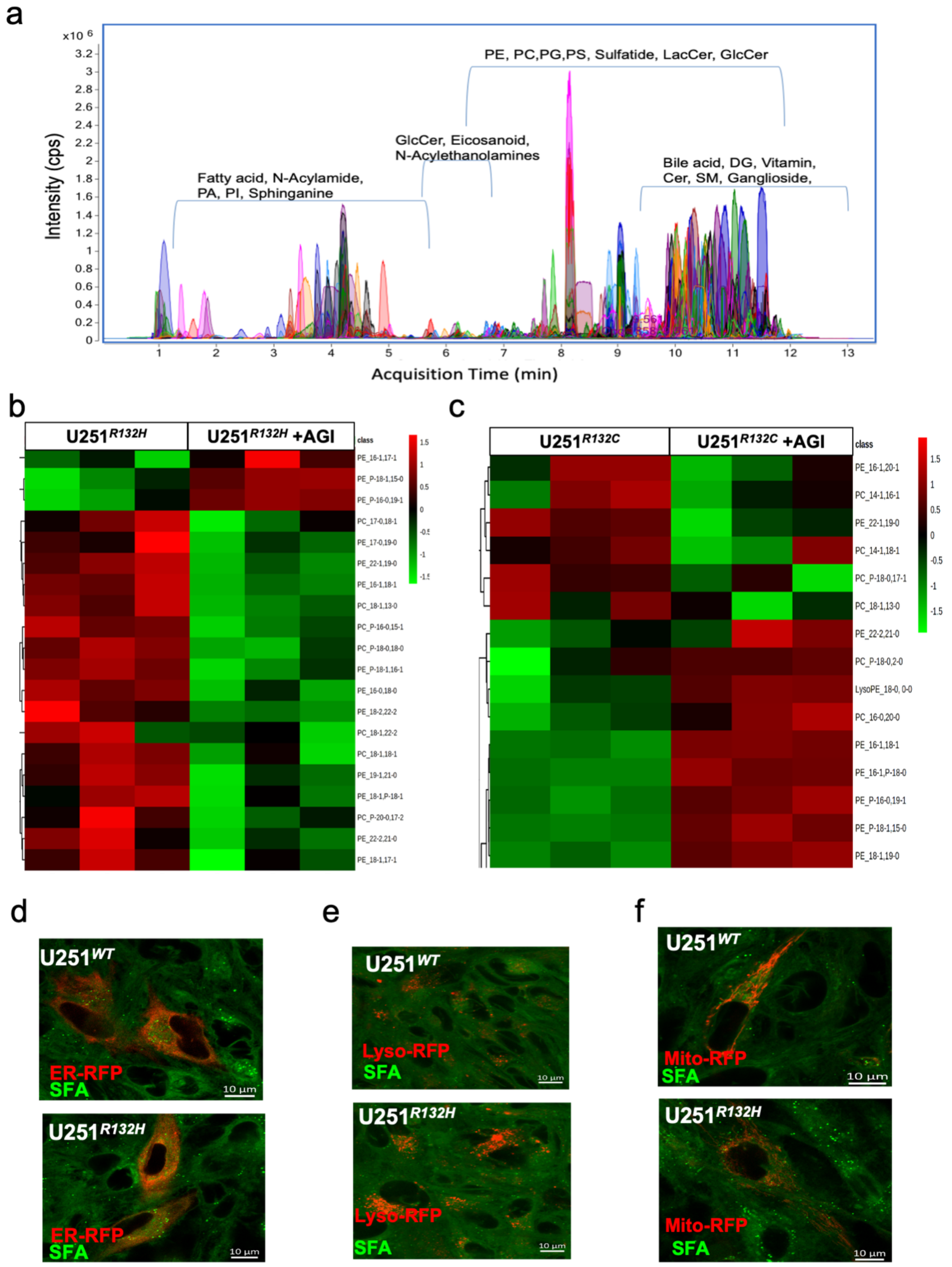

Supplementary Fig 3. **Phospholipid imbalance in ER**. **a**. Representative LC/MS chromatogram showing the classes of lipids detected with our lipidomics assay. The chromatogram was obtained from the pooled sample, which is a equal volume mixture of all the samples. The representative extracted compound chromatogram (ECC) comes from for ER-specific U251^{R132H} nonpolar lipid extract profile using CSH C18 ESI+ lipidomic assay. **b** and **c** Heatmaps of MUFA-phospholipid levels after addition of 12.5 μM AGI-5198. Heatmaps were created using MetaboAnalyst and triplicate levels of phospholipids. The data is displayed as relative levels of phospholipids for each ER sample in triplicates. Values were determined from biologically independent samples for U251^{R132C} (n=3), and U251^{R132H} (n=3) organelle. The relative abundances and lipid identities are also available in Source Data. **d-f.** Confocal Microscopy showing no signs of co-localization of SFAs (BODIPY™ FL C16, green) at ER membrane (labeled with RFP-BacMan2 (**d**) lysosome (**e**) (labeled with RFP BacMan 2) or mitochondria (**f**) (labeled with RFP BacMan 2). Scale is 10 microns. At least five images per group were taken.

Supplementary Fig. 4:

a

Patient	Type	Mutations
1 (30F)	Oligo II	IDH1 TERT, IDH1, CIC, SMARC4,
2 (44F)	AO III	TP53, FUBP1, CIC, TERT, SETD2, IDH1
3 (52F)	Oligo II	IDH1, TERT, ATRX FUBP1
4 (61F)	GBM IV	FGFR fusion, CDK4 gain, TERT, PTEN, MGMT methylated
5(49M)	GBM IV	No MGMT, TERT, PDGFRA- 10 copies

b

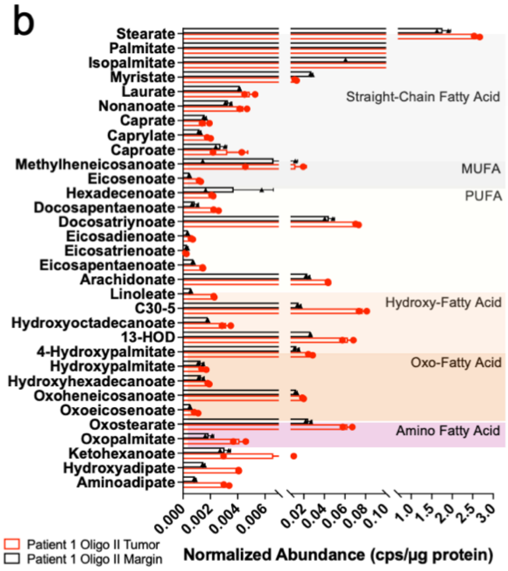

c

Patient 1 (Oligo II IDH1^{mut}) *Tissue/Margin*				
**Pathway name**	**Found**	**Ratio**	**p-value**	**FDR**
Membrane Trafficking	438/665	0.046	1.11e-16	7.89e-14
Translation	236/339	0.023	1.11e-16	7.89e-14
Axon Guidance	357/584	0.04	1.11e-16	7.89e-14

U251$^{R132H/WT}$ or U251$^{R132C/WT}$				
**Pathway name**	**Found**	**Ratio**	**p-value**	**FDR**
Translation	238/294	0.026	1.11e-16	7.61e-14
Membrane Trafficking	421/635	0.056	1.11e-16	7.61e-14
Metabolism of RNA	461/675	0.059	1.11e-16	7.61e-14

Supplementary Fig 4. Molecular characterization of patient samples and their lipidomic and proteomic analyses. a Description of histological and molecular characteristics of tissue used in this study. **b**. Relative abundances of fatty acids in tumor verus margin of Patient 1 samples. Data are shown as mean ± SEM and are representative of two technical replicates after normalization to protein content. **c** Statistics for proteomics data to highlight the pathways that are most significantly altered in Golgi apparatus of Patient 1 sample and U251 golgi cells. The tables are describing the statistics for the top 3 pathways altered based upon their p value for the three comparisons. Note that both variants were analyzed separately, however their results were similar for the top 3 pathways. Detailed pathway analysis is provided in Supplementary Tables 2-4.

Supplementary Fig. 5:

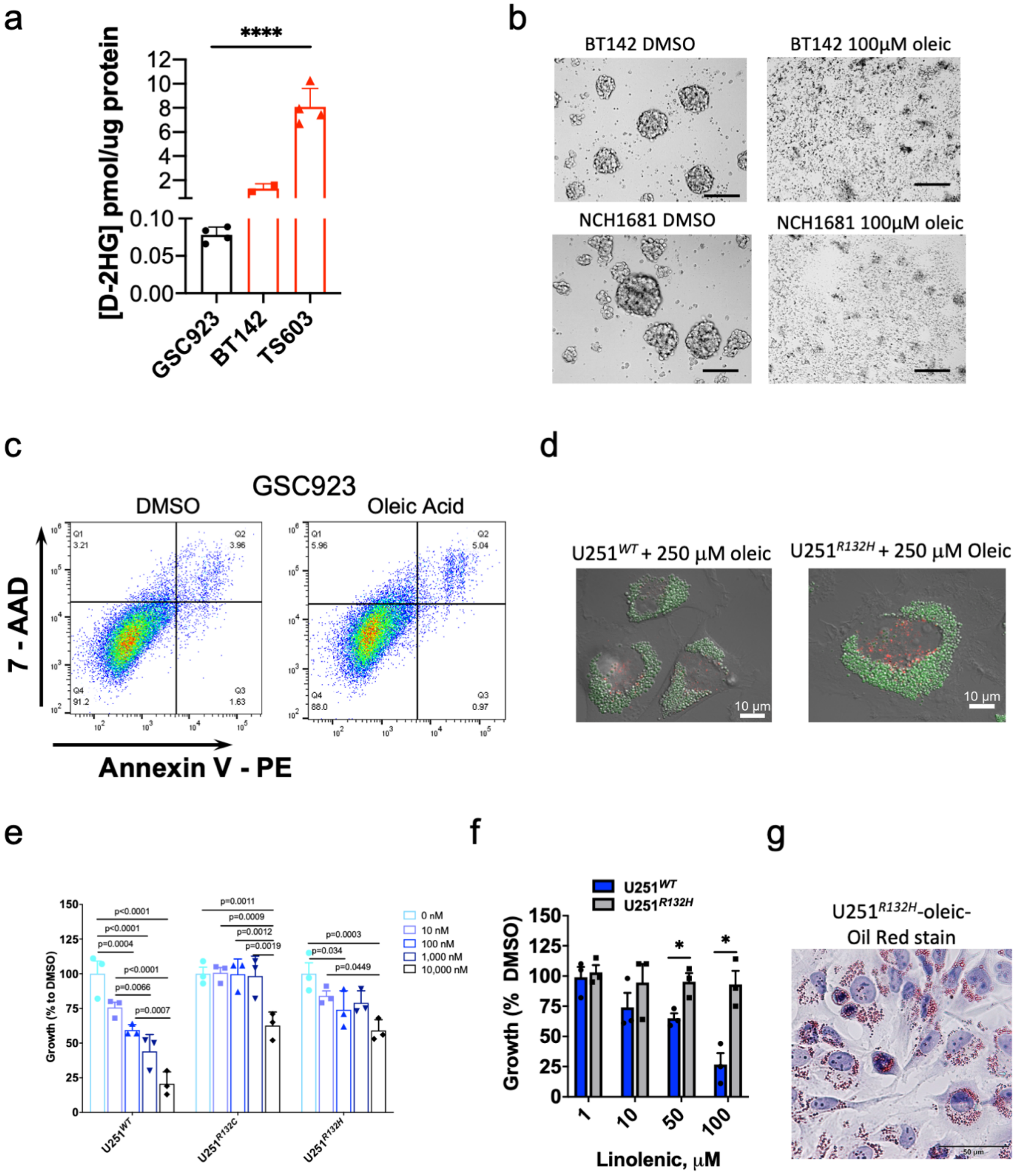

Supplementary Fig. 5. MUFA-induced cell death specific to IDH1^{mut} cells. a. Mass spectrometry-based quantification of D-2HG in the patient derived cell lines (n=4) for TS603 and GSC923 and n=2 for BT142). Data are shown as mean ± SD and are representative of two or four biological replicates. One-way Anova

followed by Tukey's test for multiple comparison of each group were conducted using GraphPad Prism 8.2.1. Statistical values are represented as: ns, not significant; *, p≤ 0.05; **p≤ 0.005; *** p≤ 0.0005; **** p≤ 0.0001 p value for GSC923 and TS603 was less than 0.0001.**b**. Addition of oleic acid leads to visible cell death as shown in the images of TS603 (upper panels), BT142 (middle panels) and NCH1681 (lower panel). Micrographs were obtained with a Leica Microscope. At least five images per group were taken. Scale bar correspond to 100 microns. **c**. Oleic acid addition does not lead to apoptosis in GSC923 cells. **d.** Addition of oleic acid led to the accumulation of lipid droplets. Scale is 50 microns. At least five images per group were taken. **e**. Inhibition of Fatty acid synthase (FASN) leads to more specific growth inhibition on $U251^{WT}$ cells. One way ANOVA with Tukey's test for multiple comparison of each group were conducted using GraphPad Prism 8.2.1 using. Statistical values are represented as numbers on the graph. Data are shown as mean ± SD and are representative of three biological replicates. **f**. Addition of linoleic acid (PUFA) affects the growth of $U251^{WT}$ cells specifically. Mutiple t-tests were conducted using GraphPad Prism 8.2.1. Values were determined from biologically independent samples for $U251^{WT}$ (n=3) and $U251^{R132H}$ (n=3) cells. Data are presented as mean values +/-SD. Statistical values are represented as: ns, not significant; *, p≤ 0.05; **p≤ 0.005; *** p≤ 0.0005; **** p≤ 0.0001. For 10 and 100 uM linoleic acid the exact p values were: 0.0207 and 0.0110, respectively. **g**. Oil red stain confirms the accumulation of neutral lipids in the lipid droplets formed by the oleic acid addition. Scale is 50 microns. At least five images per group were taken.

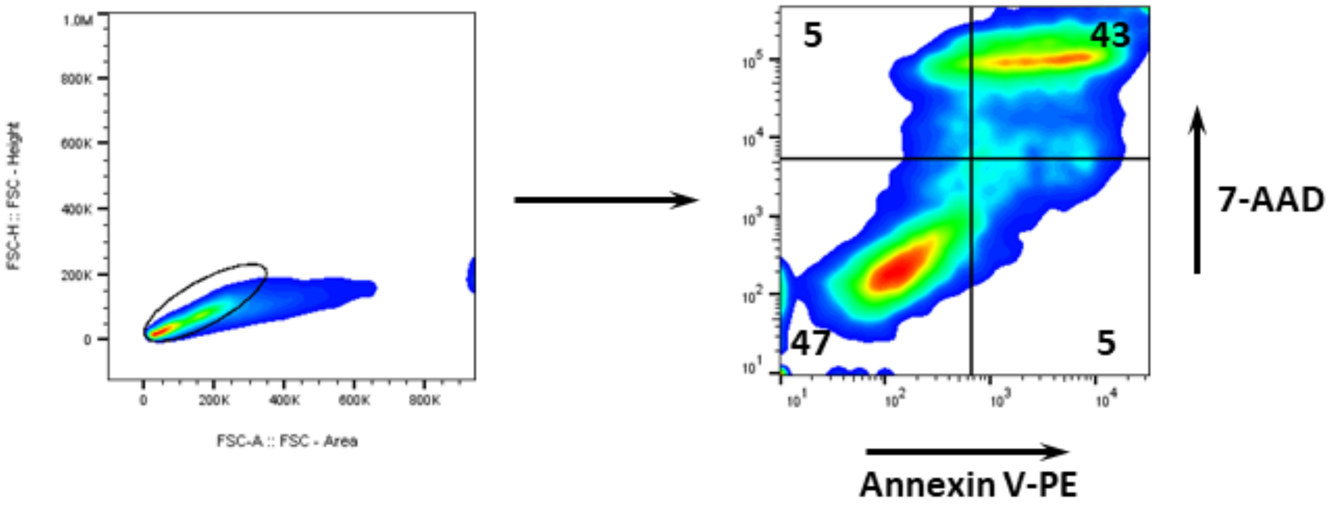

Supplementary Fig. 6. Representative flow cytometry gating strategies. This data corresponds to the TS603 cells treated with oleic acid, which is shown in Figure 6k. Doublets were excluded on forward scatter plots (FCS-H vs FSC-A) and apoptotic cells were identified with Annexin-V and 7AAD staining where Annexin-V and 7AAD double positive cells are counted as dead cells. Gates were adjusted based on unstained negative control profiles.
