# Supplementary material for: IDH1 mutations induce organelle defects via dysregulated phospholipids
Source: Nat Commun. 2021 Jan 27;12:614. doi: 10.1038/s41467-020-20752-6 (PMC7840755; doi:10.1038/s41467-020-20752-6)
Supplement: Supplementary file 2 — Reporting Summary [file 41467_2020_20752_MOESM2_ESM.pdf]

nature research

Corresponding author(s): Mioara Larion

Last updated by author(s): Nov 8, 2020

Reporting Summary

Nature Research wishes to improve the reproducibility of the work that we publish. This form provides structure for consistency and transparency in reporting. For further information on Nature Research policies, see our Editorial Policies and the Editorial Policy Checklist.

Statistics

For all statistical analyses, confirm that the following items are present in the figure legend, table legend, main text, or Methods section.

n/a	Confirmed	
☐	☒	The exact sample size (n) for each experimental group/condition, given as a discrete number and unit of measurement
☐	☒	A statement on whether measurements were taken from distinct samples or whether the same sample was measured repeatedly
☐	☒	The statistical test(s) used AND whether they are one- or two-sided<br>*Only common tests should be described solely by name; describe more complex techniques in the Methods section.*
☒	☐	A description of all covariates tested
☐	☒	A description of any assumptions or corrections, such as tests of normality and adjustment for multiple comparisons
☐	☒	A full description of the statistical parameters including central tendency (e.g. means) or other basic estimates (e.g. regression coefficient) AND variation (e.g. standard deviation) or associated estimates of uncertainty (e.g. confidence intervals)
☐	☒	For null hypothesis testing, the test statistic (e.g. F, t, r) with confidence intervals, effect sizes, degrees of freedom and P value noted<br>*Give P values as exact values whenever suitable.*
☒	☐	For Bayesian analysis, information on the choice of priors and Markov chain Monte Carlo settings
☒	☐	For hierarchical and complex designs, identification of the appropriate level for tests and full reporting of outcomes
☒	☐	Estimates of effect sizes (e.g. Cohen's d, Pearson's r), indicating how they were calculated

Our web collection on statistics for biologists contains articles on many of the points above.

Software and code

Policy information about availability of computer code

Data collection	Raman DXR2 from Thermofisher, LC/MS-QTOF 6545 from Agilent, BCAbox Software, Q Exactive, HF, Thermo Scientific, Thermo Easy nLC, Zeiss LSM880 confocal microscope, Sony SA3800 spectral analyzer, Hitachi 7600 and Hitachi 7650 TEM equipped with a 2k x 2k AMT,
Data analysis	Prism Graphpad 8.2.1 https://www.graphpad.com/scientificsoftware/prism/<br>R R Project for Statistical Computing https://www.rproject.org/<br>Metaboanalyst<br>MassHunter Quant Agilent<br>Agilent Masshunter Profinder<br>Partek Genomic Suite<br>Thermo Scientific™ OMNIC™xi Software<br>BCAbox 1.0 Software<br>Proteome Discoverer 2.2 software (Thermo Scientific, CA)<br>Percolator - omicX<br>Flowjo™10.6.1<br>AMT software<br>Image J 1.52a

For manuscripts utilizing custom algorithms or software that are central to the research but not yet described in published literature, software must be made available to editors and reviewers. We strongly encourage code deposition in a community repository (e.g. GitHub). See the Nature Research guidelines for submitting code & software for further information.

Data

Policy information about availability of data

All manuscripts must include a data availability statement. This statement should provide the following information, where applicable:

- Accession codes, unique identifiers, or web links for publicly available datasets
- A list of figures that have associated raw data
- A description of any restrictions on data availability

The datasets generated during and/or analyzed during the current study are available. Source data contains all the raw data reported in this manuscript. Lipidomics and proteomics data are deposited and the links below. Data supporting the findings of this work are available within the paper and its Supplementary Information files. A reporting summary for this article is available as a Supplementary Information file. The metabolic datasets generated and analyzed during the current study are available from the corresponding author upon request and have been deposited in Metabolights database under the accession numbers: www.ebi.ac.uk/metabolights/MTBLS1974; www.ebi.ac.uk/metabolights/MTBLS1973; www.ebi.ac.uk/metabolights/MTBLS1967.
The proteomics dataset generated in this study has been deposited in to MassIVE under the accession number: MSV000085841. Reactome database was used for proteomics analysis. Source data are provided with this paper.
All the figures have raw data.

Field-specific reporting

Please select the one below that is the best fit for your research. If you are not sure, read the appropriate sections before making your selection.

☒ Life sciences ☐ Behavioural & social sciences ☐ Ecological, evolutionary & environmental sciences

For a reference copy of the document with all sections, see nature.com/documents/nr-reporting-summary-flat.pdf

Life sciences study design

All studies must disclose on these points even when the disclosure is negative.

Sample size	No statistical method was used to predetermine sample size.
Data exclusions	Compound features containing zero values were eliminated to reduce bias. This exclusion criteria was pre-established.
Replication	Unless explicitly stated, data shown were obtained from at least 3 biological independent experiments. All attempts were successful at replication although there were some variations of values. For representative images, each experiment was successfully repeated at least three times under similar conditions.
Randomization	We performed randomization of samples before acquisition on all of the experiments.
Blinding	Blinding was not relevant for this study because we did not conduct animal or clinical studies. However, lipidomics and organelle analyses were performed in a blinded fashion.

Reporting for specific materials, systems and methods

We require information from authors about some types of materials, experimental systems and methods used in many studies. Here, indicate whether each material, system or method listed is relevant to your study. If you are not sure if a list item applies to your research, read the appropriate section before selecting a response.

Materials & experimental systems

n/a	Involved in the study	
☐	☒	Antibodies
☐	☒	Eukaryotic cell lines
☒	☐	Palaeontology and archaeology
☒	☐	Animals and other organisms
☐	☒	Human research participants
☒	☐	Clinical data
☒	☐	Dual use research of concern

Methods

n/a	Involved in the study	
☒	☐	ChIP-seq
☐	☒	Flow cytometry
☒	☐	MRI-based neuroimaging

Antibodies

Antibodies used	SCD-1 Abcam ab19862<br>SCD-5 Abcam ab130958<br>FASN Abcam ab218306<br>tubulin Abcam ab15568

	b-actin (D6A8) Rabbit Cell signaling Technology 8457<br>Anti-Rabbit IgG HRP-linked Cell signaling Technology 7074
Validation	https://www.abcam.com/scd1-antibody-cde10-ab19862.htmlhttps://www.abcam.com/fatty-acid-synthase-antibody-ab22759.html<br>https://www.abcam.com/beta-tubulin-antibody-ab15568.html<br>https://www.cellsignal.com/products/primary-antibodies/b-actin-d6a8-rabbit-mab/8457<br>https://www.abcam.com/scd5-antibody-ab130958.html<br>For ab19862's knockout validation, please see this Western Blot image.<br>https://www.abcam.com/scd1-antibody-cde10-ab19862.html#description_images_1<br>ab130958 was validated in Human fetal heart lysate, and this data is available here.<br>https://www.abcam.com/scd5-antibody-ab130958.html#description_images_1<br>https://www.cellsignal.com/products/secondary-antibodies/anti-rabbit-igg-hrp-linked-antibody/7074?_=1604973969470&Ntt=HRP%20secondary%20anti&tahead=true

Eukaryotic cell lines

Policy information about cell lines

Cell line source(s)	U251 was purchased from Sigma-Aldrich (Cat # 09063001)<br>TS603 MSKCC N/A<br>BT142 ATCC ATCC® ACS-1018™<br>GSC827 Neuro-Oncology Branch<br>GSC923 Neuro-Oncology Branch<br>NCH1681 University of Heidelberg
Authentication	our cell lines were analyzed via Whole exon sequencing, DNA methylation and RNA sequencing
Mycoplasma contamination	we conduced MTBM testing of cell lines and we found no mycoplasma in them.
Commonly misidentified lines<br>(See ICLAC register)	No misindetified cell lines were used.

Human research participants

Policy information about studies involving human research participants

Population characteristics	*Describe the covariate-relevant population characteristics of the human research participants (e.g. age, gender, genotypic information, past and current diagnosis and treatment categories). If you filled out the behavioural & social sciences study design questions and have nothing to add here, write "See above."*
Recruitment	*Describe how participants were recruited. Outline any potential self-selection bias or other biases that may be present and how these are likely to impact results.*
Ethics oversight	*Identify the organization(s) that approved the study protocol.*

Note that full information on the approval of the study protocol must also be provided in the manuscript.

Flow Cytometry

Plots

Confirm that:

- [x] The axis labels state the marker and fluorochrome used (e.g. CD4-FITC).
- [x] The axis scales are clearly visible. Include numbers along axes only for bottom left plot of group (a 'group' is an analysis of identical markers).
- [x] All plots are contour plots with outliers or pseudocolor plots.
- [ ] A numerical value for number of cells or percentage (with statistics) is provided.

Methodology

Sample preparation	Apoptosis was assessed using PE Annexin V Apoptosis<br>Detection Kit I (BD Biosciences) and analyzed by flow cytometry. Briefly, cells were plated into 6-well culture<br>dishes (1×106 cells/well) for 24 hrs. prior to the addition of oleic acid (Sigma Cat#75090) at a concentration<br>of 150 µM. Following 24 hrs incubation with oleic acid the percentage of apoptotic cells was determined by<br>the annexin V-PE/7-AAD assay following manufacturer's instructions. Fluorescence of the cells was<br>immediately determined by a Sony SA3800 spectral analyzer. Control samples were prepared for all single dye staining.
Instrument	Sony SA3800 spectral analyzer
Software	Flowjo™

Cell population abundance	between 3- 9 percent of apoptotic cells in IDHWT and 43% in IDHmut cells treated with oleic acid. IDHmut DMSO had only 11% apoptotic cells
Gating strategy	Doublets were excluded based on the forward scattering (FSC-H vs FSC-A).

☒ Tick this box to confirm that a figure exemplifying the gating strategy is provided in the Supplementary Information.
